# A gradient tree boosting and network propagation derived pan-cancer survival network of the tumor microenvironment

**DOI:** 10.1016/j.isci.2021.103617

**Published:** 2021-12-11

**Authors:** Kristina Thedinga, Ralf Herwig

**Affiliations:** 1 Department of Computational Molecular Biology, Max-Planck-Institute for Molecular Genetics, Ihnestrasse 63-73, 14195 Berlin, Germany

**Keywords:** Bioinformatics, Cancer systems biology, Mathematical biosciences

## Abstract

Predicting cancer survival from molecular data is an important aspect of biomedical research because it allows quantifying patient risks and thus individualizing therapy. We introduce XGBoost tree ensemble learning to predict survival from transcriptome data of 8,024 patients from 25 different cancer types and show highly competitive performance with state-of-the-art methods. To further improve plausibility of the machine learning approach we conducted two additional steps. In the first step, we applied pan-cancer training and showed that it substantially improves prognosis compared with cancer subtype-specific training. In the second step, we applied network propagation and inferred a pan-cancer survival network consisting of 103 genes. This network highlights cross-cohort features and is predictive for the tumor microenvironment and immune status of the patients. Our work demonstrates that pan-cancer learning combined with network propagation generalizes over multiple cancer types and identifies biologically plausible features that can serve as biomarkers for monitoring cancer survival.

## Highlights

• Highly performing cancer survival prediction with XGBoost
• Pan-cancer training outperforms single-cohort training
• Combined approach consisting of machine learning and network propagation
• Tumor microenvironment is most strongly involved in cancer survival prediction

## Graphical abstract

**Figure:**
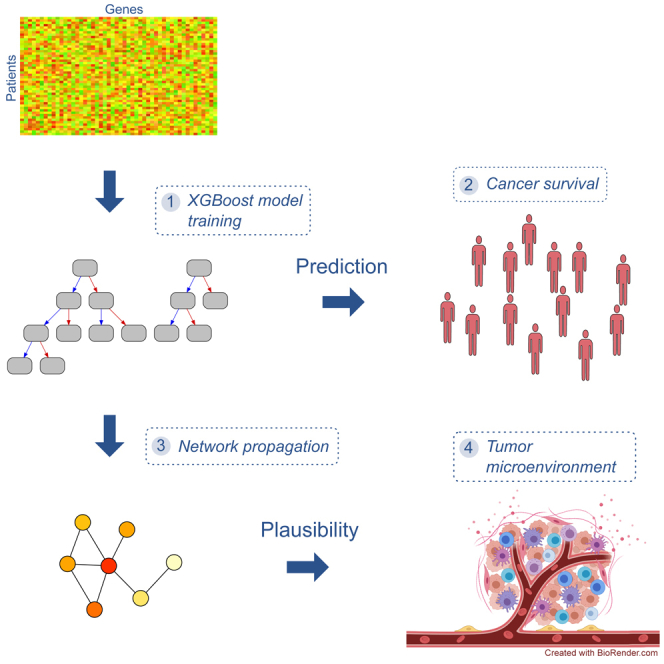

## Introduction

Patient survival is the ultimate goal of cancer therapy and predicting patient survival from the molecular features of the individual tumor is an important computational task that has implications for tumor progression, therapy, and patient care (Hoadley et al., 2018). Large population studies have shown that cancer survival is a multi-factorial problem and varies broadly between cancer types (Allemani et al., 2015, 2018). This has given rise to the identification of numerous gene expression signatures specific for given cancer sub-types or treatments; however, such signatures are often hardly reproducible (Venet et al., 2011) and depend on the statistical approach, the individual patient cohorts used, and even the normalization of the data (Patil et al., 2015). In addition to gene expression signatures, it has recently also been reported for mutation-based signatures that there is a lack of biological cause and interpretation (Alexandrov et al., 2020; Kim et al., 2020). This is due to rather small sample sizes of subtype-specific patient cohorts and high inter-patient heterogeneity of the molecular features of the patients within the cancer subtypes.

To overcome these issues, pan-cancer approaches have been conducted (The ICGC/TCGA Pan-Cancer Analysis of Whole Genomes Consortium, 2020), and these studies have indeed shown that there are common regulatory mechanisms and features that are shared by patients of different cancer sub-types, for example, on the level of signaling pathways (Sanchez-Vega et al., 2018). Additionally, it has been shown that the immune status of the patients is an important factor in cancer survival (Thorsson et al., 2018). Furthermore, cancer onset and progression are correlated with age, and many of the hallmarks of cancer are shared in aging (Aunan et al., 2017). In particular, there is interplay between aging cells in the microenvironment and cancer cells that may impact tumor metastasis and therapy responses (Fane and Weeraratna, 2020). This suggests that there might be common patterns for survival prognosis across different cancer types and only recently survival prognoses based on pan-cancer approaches have been developed (Cheerla and Gevaert, 2019; Kim et al., 2020).

Computational approaches predicting survival from patients’ molecular data have been conducted based on different machine learning techniques (Kourou et al., 2015). For example, Ishwaran et al. (2008) developed a method for right-censored data that is based on random forests and uses ensembles of decision trees for survival prediction, while Khan and Zubek (2008) adapted support vector machines to predict patient survival. More recently, Li et al. (2016) introduced a multitask learning formulation for survival prediction, where the survival time is predicted by estimating the patients’ survival status at certain time intervals over the study period. Other recent methods for predicting cancer patient survival include Cox-nnet (Ching et al., 2018), which uses a neural network in combination with Cox regression as an output layer, and DeepSurv (Katzman et al., 2018), a deep neural network survival prediction method that besides predicting survival also models the relationship between patient covariates and treatment effectiveness in order to make personalized treatment recommendations. While these approaches are mainly gene-centered, it has been argued that they provide limited understanding of mechanistic cancer survival processes. Therefore, machine learning approaches have been developed that incorporate pathway and network features directly in the learning process so that derived features highlight potential molecular mechanisms of cancer survival such as the Path2Surv method (Dereli et al., 2019).

A key computational concern of machine learning is interpretability, i.e., the ability to recall how the algorithm has come to its decisions on global and local levels. This definition has recently been expanded by defining interpretability as the “extraction of relevant knowledge”, i.e., to also generate plausible results (Murdoch et al., 2019). Because complex machine learning methods, in particular tree ensemble methods and (deep) neural networks, have generally a lower level of interpretability than, for example, simple decision trees or linear regression approaches, this might necessitate additional methods that add plausibility in the context of the concrete data domain, such as the incorporation of biological networks. An approach to investigate biological mechanisms from molecular data is network propagation (Cowen et al., 2017). Network propagation allows combining experimental data with molecular interaction networks, such that the topology of the network is used to propagate the data effects throughout the network, and by that amplifying and functionally interpreting the experimental data. This approach covers a wide range of data domains and has been applied, for example, to associate genetic variants with phenotypic disease traits (Barel and Herwig, 2020; Leiserson et al., 2015).

In this work we combine state-of-the-art tree ensemble learning and network propagation in order to derive and characterize a pan-cancer molecular signature for survival prognosis that is both computationally and biologically interpretable. We have analyzed gene expression data from the TCGA cancer cohorts summarizing to 8,024 patients from 25 different cancer cohorts (Liu et al., 2018). Our machine learning approach is based on the XGBoost tree ensemble method (Chen and Guestrin, 2016), which has been shown to provide good performance in many application domains, for instance, in diagnosing chronic kidney disease (Ogunleye and Wang, 2020) or identifying patients with epilepsy based on their cerebral activity (Torlay et al., 2017). We compare the XGBoost method against four other state-of-the-art methods and show highly competitive performance in single-cancer cohort survival prediction. From the comparison, we observed that age, in particular, is a confounding cross-cohort factor and that overall survival prediction performance of all methods decreased with the age of the patients. This led us to question whether there is a common signature of cancer survival in the patients that is independent of the particular cancer type. We thus set up a workflow for pan-cancer training and showed increased prognostic performance compared to single-cancer prediction. Features dominant for pan-cancer prediction were further quantified and network propagation was carried out with the trained feature importance weights in order to identify molecular mechanisms related to pan-cancer survival. Network propagation identified a subnetwork consisting of 103 genes that is strongly associated with the tumor microenvironment (TME) and regulated by important factors of the TME such as *TNF*, *TP53*, and *STAT3*. Furthermore, we can show that the immune status of the patients can partially be recovered by the survival network signature.

Our study shows that gradient tree boosting can be efficiently applied to pan-cancer survival prediction and that the combination of machine learning and network propagation can identify biologically meaningful subnetworks that highlight the importance of the TME for patient survival. The combination of machine learning and subsequent feature-informed network propagation is fairly generic and can be generalized to other disease domains and biological research questions.

## Results

### XGBoost gradient boosting predicts survival of cancer patients

**Figure 1:**
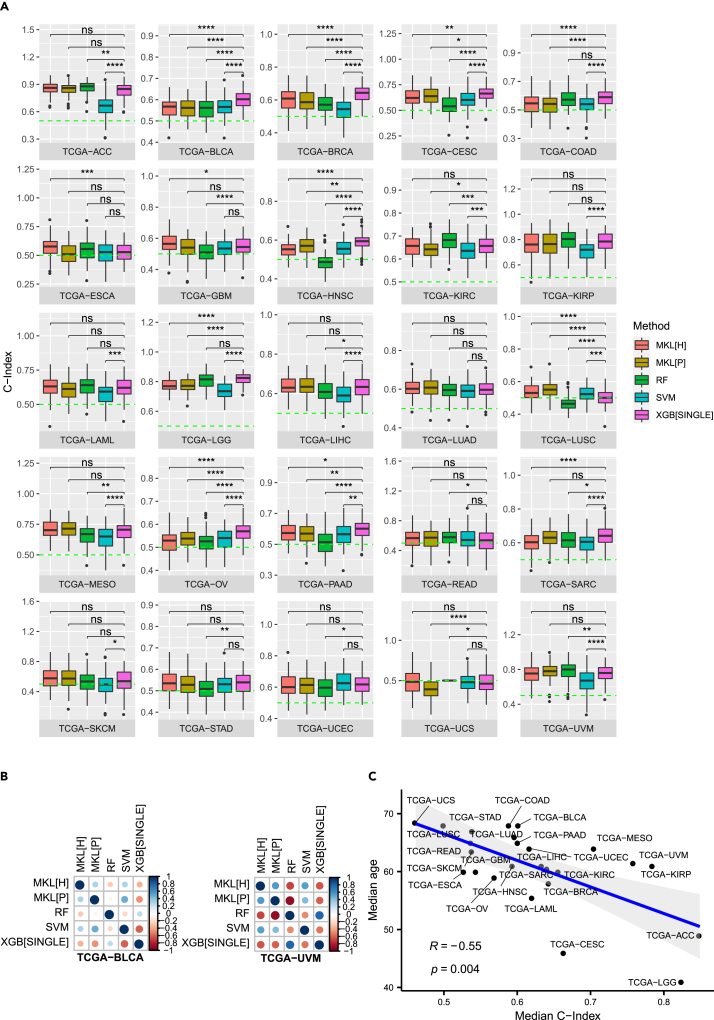
Single-cohort prediction performances (A) C-Index boxplots over 100 replications of model training for random survival forest (RF), survival SVM (SVM), the Path2Surv multiple-kernel learning on the Hallmark gene sets (MKL[H]) and the Pathway Interaction Database (MKL[P]), and the single-cohort XGBoost method (XGB[SINGLE]) on 25 different TCGA cancer cohorts. Mean C-Indices were compared with Wilcoxon’s unpaired rank-sum test and significance levels are defined as ns: p>0.05, ∗:p≤0.05, ∗∗:p≤0.01, ∗∗∗:p≤0.001, ∗∗∗∗:p≤0.0001. (B) Spearman correlations between predictions of the different methods for test patients from the cohorts TCGA-BLCA (left) and TCGA-UVM (right). Larger circles correspond to a greater correlation, blue indicates a positive correlation and red indicates a negative correlation. (C) Spearman correlation (*R*) between median C-Indices of single-cohort XGBoost predictions and median ages for 25 different TCGA cohorts. The blue line shows the linear regression fit to the data and the gray area indicates the 95% confidence interval.

In order to assess the ability of the XGBoost method to predict survival from gene expression data of cancer patients, we compared the single-cohort XGBoost method against three well-established survival prediction methods, namely random survival forest (Ishwaran et al., 2008), survival support vector machine (Khan and Zubek, 2008; Shivaswamy et al., 2007), and the Path2Surv multiple-kernel learning method (Dereli et al., 2019) (cf. STAR Methods). Figure 1A shows the prediction performances of random survival forest (RF), survival SVM (SVM), the Path2Surv multiple-kernel learning method on two different pathway/gene set collections (MKL[H] on the Hallmark gene sets (Liberzon et al., 2015) and MKL[P] on the Pathway Interaction Database (PID) (Schaefer et al., 2009)), and the single-cohort XGBoost method (XGB[SINGLE]) on 25 different TCGA cancer cohorts (Table S1). These cohorts represent a wide spectrum of the human organ system (Parris, 2020). For ten of the cohorts (TCGA-BLCA, TCGA-BRCA, TCGA-CESC, TCGA-COAD, TCGA-HNSC, TCGA-LGG, TGCA-OV, TGCA-PAAD, TCGA-SARC, and TCGA-STAD) single-cohort XGBoost showed the best median performance, while RF performed best for seven cohorts (TCGA-ACC, TCGA-KIRC, TCGA-KIRP, TCGA-LAML, TCGA-READ, TCGA-UCS, and TCGA-UVM). The Path2Surv method outperformed the other methods in four (TCGA-LIHC, TCGA-LUAD, TCGA-LUSC, and TCGA-MESO) and three (TCGA-ESCA, TCGA-GBM, and TCGA-SKCM) cohorts for MKL[P] and MKL[H], respectively, while SVM could achieve the best median performance in only one cohort (TCGA-UCEC). Overall, single-cohort XGBoost significantly outperformed RF for 13, SVM for 17, MKL[H] for 9 and MKL[P] for 10 out of 25 TCGA cohorts.

For two of the 25 cohorts (TCGA-BLCA and TCGA-UVM) we also assessed how well the survival predictions of the different methods were correlated. To this end, for each of the two selected cohorts, we trained and tested all methods on the same train-test-split of patients and computed the Spearman correlation between survival predictions for the test patients. Since Path2Surv (MKL[H] and MKL[P]) and SVM predict survival times, while RF and XGBoost predict risk scores, where a higher risk score corresponds to a shorter predicted survival, predictions of Path2Surv and SVM are expected to be negatively correlated to predictions of RF and XGBoost. Figure 1B shows the correlation values between predictions of the different methods. As expected, predictions of the same metric (either survival time or risk score) are positively correlated, while predictions of survival time are negatively correlated to risk score predictions in most cases. However, correlation strengths differ between methods and among the analyzed cohorts. The survival predictions of the different methods seem to be more highly correlated in TCGA-UVM than in TCGA-BLCA. In TCGA-UVM predictions are most correlated between MKL[P] and RF and between XGB[SINGLE] and RF. In TCGA-BLCA, the highest Spearman correlation can be observed between XGB[SINGLE] and SVM, while the other correlations are rather weak.

Age is the predominant indicator of tumor development and the probability of developing cancer increases from 3.4% in the time span from birth to 49 years of age (male; female 5.5%) to 32.2% in the time span above 70 years of age (female 26%) (Siegel et al., 2018). We observed that survival prediction performance to a certain degree is dependent on the age distribution of the cohort under study. For example, predictions for TCGA-ACC and TCGA-LGG were exceptionally good (median C-Index > 0.8 with the XGBoost single-cohort method) and ages of patients in these cohorts were consistently low with an overall median of 49 and 41 years. This overall median increases to 61 and 61.5 years with TCGA-KIRP and TCGA-UVM and, likewise, the prediction performance drops below 0.8 in both cohorts. All prediction methods performed bad for the TCGA-UCS cohort (∼0.5 prediction performance) with a median age of 68.5 years. In fact, the median C-Indices of the predictions made by the single-cohort XGBoost approach and the median age of the correspondent cohort are negatively correlated (Spearman R = −0.55, p = 0.004; Figure 1C), meaning that prognosis performances tend to be better for cancers of younger patients. This indicates age-specific gene expression signatures in the datasets under study that are easier to be resolved by machine learning methods in younger, and presumably more intact, states.

### Pan-cancer training improves over single-cohort training

**Figure 2:**
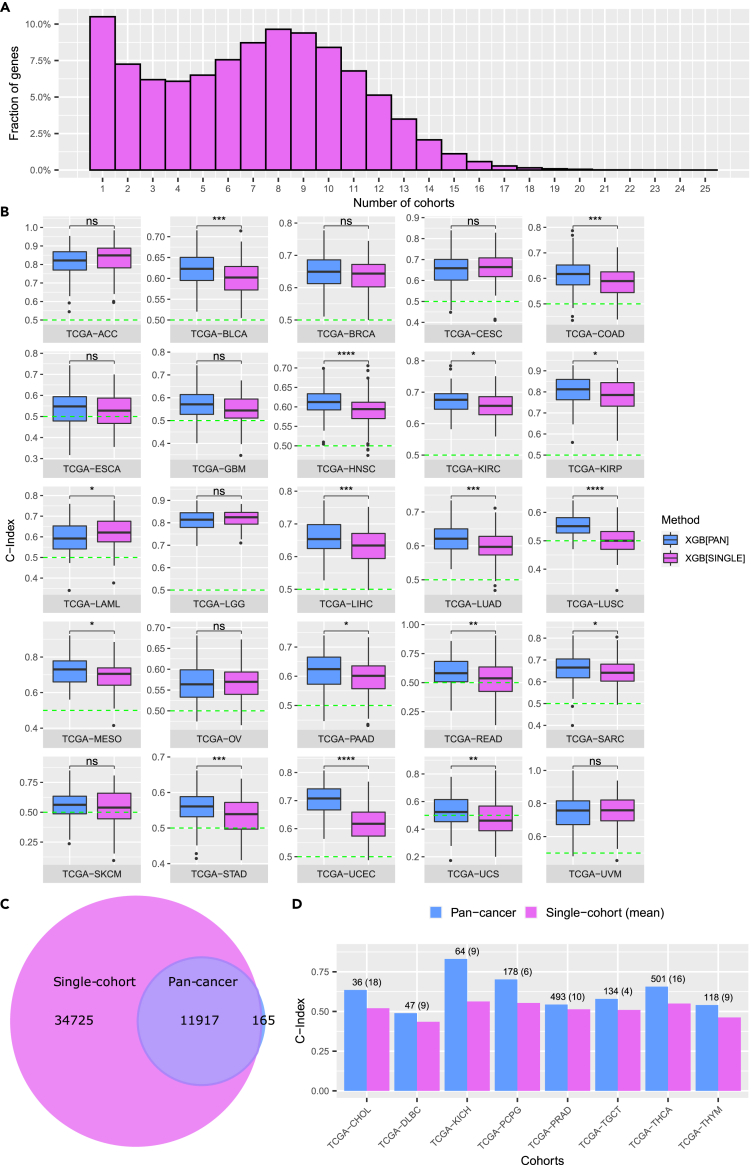
Pan-cancer XGBoost training improves over single-cohort training (A) Histogram depicting fractions of gene features shared over different numbers of training cohorts (x axis: number of TCGA cohorts a gene feature is shared over; y axis: fraction of all 46,642 genes used in at least one single-cohort model). (B) Prediction performances of the single-cohort XGBoost method (XGB[SINGLE]) and the pan-cancer XGBoost method (XGB[PAN]) on 25 different TCGA cancer cohorts, depicted as C-Index boxplots over 100 replications of model training. Mean C-Indices were compared with the Wilcoxon unpaired rank-sum test and significance levels are defined as ns: p>0.05, ∗:p≤0.05, ∗∗:p≤0.01, ∗∗∗:p≤0.001, ∗∗∗∗:p≤0.0001. See also Figure S5. (C) Venn-diagram comparing features used for prediction in the single-cohort XGBoost method (pink) with those selected in the pan-cancer XGBoost method (blue). See also Figure S1. (D) Prediction performances (C-Indices) of single-cohort XGBoost (pink) and pan-cancer XGBoost (blue) for eight new cancer cohorts (not used in model training). For the single-cohort method, the mean C-Index over all 25 models trained on different TCGA cohorts is shown.

We next investigated the gene expression features that were used by the single-cohort XGBoost method to predict survival in the different TCGA cancer cohorts. XGBoost has several built-in types of importance values that are computed during the training process. For each cohort and each of the training replications we computed the ‘gain’ of each gene expression feature which refers to the improvement in the used evaluation metric that the particular feature gives to the different branches it is on (cf. STAR Methods). Since the single-cohort XGBoost method was trained for 100 replications on each cohort with varying splits of training and test data, the genes selected in the feature selection step as well as the final feature importance values can vary among the replications and even more so among the different cancer cohorts depending on the patients in the training set. Across all 25 cohorts and all 100 replications per cohort, there is a total of 46,642 different RNA molecules that were captured by the TCGA data analysis pipeline (Gao et al., 2019) and used for prognosis by the XGBoost method, including protein coding genes, processed pseudogenes (Cheetham et al., 2020), and long non-coding RNAs (Statello et al., 2021), among others. In Figure 2A, we analyzed to what extent these gene features were shared between the different cohorts. It can be observed that most genes gain feature importance for a smaller number of cohorts (<10) while there are only a very small number of genes that are among the important features for a larger fraction of the cohorts (>15). This accounts for cancer subtype differences, tissue-specificity as well as inter-patient heterogeneity.

To overcome this heterogeneity across subtypes and patients and to retrieve features with more general importance, we thus trained the XGBoost method on a joint dataset combining the different cohorts (pan-cancer training; cf. STAR Methods) by using pooled gene expression data from all 25 cancer cohorts instead of training a separate model for each cohort. Figure 2B compares the C-Indices over 100 replications between the pan-cancer and single-cohort XGBoost versions. For 15 out of the 25 cohorts, pan-cancer training significantly improved the method’s prediction performance in terms of C-Index, and for nine cohorts performance was comparably good for both versions. Only in one cohort, namely TCGA-LAML, the prediction performance of the pan-cancer-trained XGBoost method was significantly worse than single-cohort-trained XGBoost. When comparing the features selected in the single-cohort training procedures with those selected in the pan-cancer version of the XGBoost method (Figure 2C), it becomes apparent that the vast majority (98.6%) of genes selected as important features in at least one of the 100 replications of the pan-cancer training were also among the important features of the single-cohort approach. However, while for the single-cohort XGBoost approach the set of important features comprises a total of 46,642 different genes, the pan-cancer approach results in a 74% reduction and identifies only 12,082 genes as important features (Figure 2C). Additionally, the type of features that are of importance change (Figure S1): While in the single-cohort approach 40.4% of the important features represent protein coding genes, 25.0% represent lncRNAs and 15.8% processed pseudogenes, in the pan-cancer approach we observe a shift toward protein coding genes (56.5%) and a reduction of lncRNAs (20.7%) and processed pseudogenes (11.9%). This shift might be driven by the tissue specificity of lncRNAs and the rather patient-specific nature of mRNA retrotransposition leading to less feature importance in the pan-cancer training.

Another benefit of pan-cancer training is that the selected features are not specific to a particular cancer type and can be extrapolated to predict survival in yet unseen patient cohorts. We challenged this claim and additionally tested the prediction performance of the pan-cancer and single-cohort trained models on eight additional TCGA cohorts, which had previously been excluded because the number of uncensored patients in these cohorts was too small (cf. STAR Methods; Table S1). Figure 2D shows the C-Indices of the predicted survival on these eight cohorts. Since there are 25 different single-cohort models (each trained on one TCGA cohort), but only one pan-cancer model trained on all cohorts jointly, we compared the prediction performance of the pan-cancer model to the mean performance of the 25 single-cohort models. For all eight cohorts, the C-Index of the pan-cancer prediction was better than the mean C-Index of the single-cohort models, and for seven cohorts, the C-Index of the pan-cancer XGBoost method was greater than 0.5, even though the entire cancer types of the test patients had not been seen in model training. This supports the hypothesis that the genes identified in the pan-cancer XGBoost approach indeed have higher predictive power for patient survival across yet unseen cancer types than the single-cohort approaches.

### Pan-cancer feature importance weights identify genes with high biological plausibility

**Figure 3:**
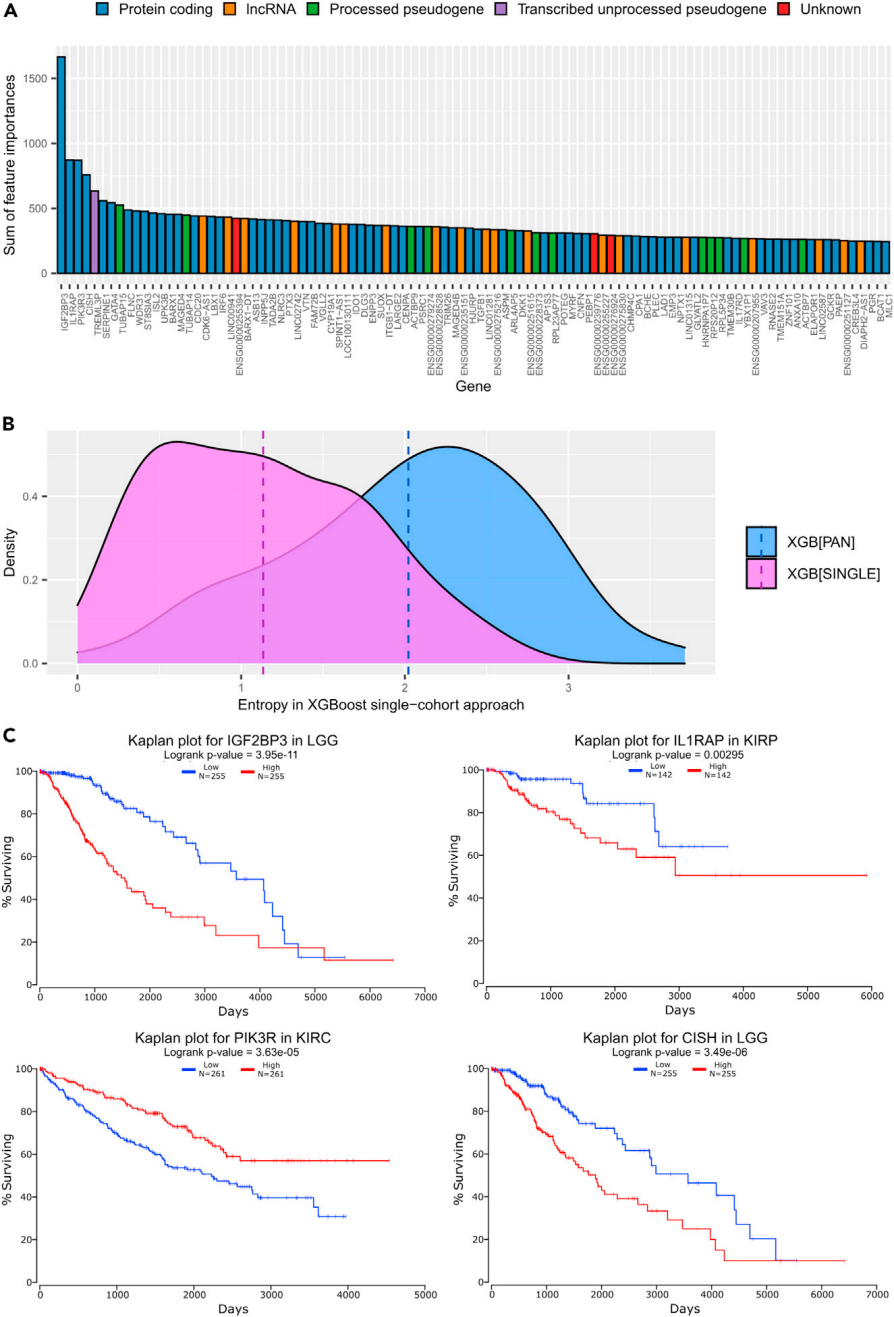
Pan-cancer features are biologically plausible (A) Weight distribution for the 100 genes with the highest feature importance (sums of feature importance scores over 100 model replications) for pan-cancer XGBoost training (gene identifiers that did not map to a Hugo symbol are named with their Ensembl identifiers). The different colors indicate gene types (blue: protein coding, orange: lncRNA, green: processed pseudogenes, purple: transcribed unprocessed pseudogene, red: gene type unknown). These genes types were obtained using the MyGene Python package (version 3.1, http://mygene.info) (Wu et al., 2013; Xin et al., 2016). See also Figure S3. (B) Comparison of entropy distributions between the top 100 genes with the highest feature importance (feature importance is measured as sums of feature importance scores over 100 model replications) from the single-cohort approach and the pan-cancer approach (mean entropies are indicated as dashed lines). The entropy measure (xaxis) is based on the genes used in the single-cohort approach (cf. STAR Methods). The density of the entropy distribution is displayed on the yaxis. (C) Kaplan-Meier plots for the four most important gene features from pan-cancer XGBoost and the cancer type with the lowest FDR-corrected p value in Cox regression, respectively. As a cutoff for gene expression, the 50^th^ percentile was selected. Cox regression data and Kaplan-Meier plots were retrieved from OncoLnc (Anaya, 2016). See also Figure S2.

We next explored the biological plausibility of the genes identified as important survival features in the pan-cancer XGBoost approach. We first investigated the distribution of summarized feature importance weights over all 100 replications of the pan-cancer training procedure. Figure 3A shows the weight distribution for the 100 genes with the highest weights, where Ensembl gene identifiers were converted to HUGO symbols with the MyGene Python package (version 3.1, http://mygene.info) (Wu et al., 2013; Xin et al., 2016) and identifiers that did not map to a HUGO symbol are named with their Ensembl gene identifier. It is noticeable that a few genes, and in particular the gene *IGF2BP3* (Insulin Like Growth Factor 2 MRNA Binding Protein 3), have especially high feature importance weights, indicating a high prognostic potential. Indeed, *IGF2BP3* has been found to be overexpressed in various cancers and has been associated with metastasis and poor survival in a number of cancer types (Mancarella and Scotlandi, 2020) including colon cancer (Lochhead et al., 2012).

In order to judge whether the genes identified as important features in the pan-cancer approach could be prognostic for several cancer types we applied an entropy score derived from their importance values in the single-cohort training models (cf. STAR Methods). For each of the single cohorts we summed the gene feature importance scores over the 100 different training repetitions to obtain a feature importance weight for each gene in each cohort. Genes have a high entropy score if they have similar feature importance weights in many different cohorts and a low entropy score if they have feature importance in only a few cohorts (range [0, 4.64]). Figure 3B compares the distributions of the entropy scores of the top 100 genes from the single-cohort approach and the top 100 genes from the pan-cancer approach. The top 100 pan-cancer genes had a significantly higher entropy than the top 100 genes from the single-cohort approach (p = 1.145 × 10^−14^ in a one-sided Wilcoxon unpaired rank-sum test), meaning the pan-cancer method generalizes better over different cancer types than the single-cohort method.

To validate the association of these pan-cancer genes with survival, we analyzed the four top-scoring protein-coding genes (*IGF2BP3*, *IL1RAP*, *PIK3R3*, and *CISH*) in more detail by querying OncoLnc (Anaya, 2016), a tool that provides Cox regression analyses as well as Kaplan-Meier survival plots on TCGA gene expression data for different cancer types. Figure 3C displays Kaplan-Meier plots of these four genes for the cancer type with the lowest FDR-corrected p value in the Cox regression, respectively. For example, *IGF2BP3* has the highest predictive performance in survival for LGG (FDR = 3.59 × 10^−9^ in Cox regression), while *IL1RAP*, *PIK3R3*, and *CISH* are most predictive for KIRP, KIRC, and LGG, respectively. Additionally, *IGF2BP3*, *IL1RAP*, *PIK3R3*, and *CISH* have significant survival performance (FDR <0.05 in Cox regression) for four (KIRP, KIRC, LUAD and PAAD), two (LGG and PAAD), two (LGG and HNSC), and four (LUAD, LIHC, KIRP, and KIRC) other cohorts, respectively. Kaplan-Meier plots for these cohorts can be found in the Supplemental information (Figure S2). For generating the Kaplan-Meier plots in Figure 3C, the patients were first split into two groups by expression of the respective gene (i.e., *IGF2BP3*, *IL1RAP*, *PIK3R3*, or *CISH*). As a cutoff, we selected the 50^th^ percentile to ensure that both groups had equal sizes and all patients were included in the Kaplan-Meier analysis. To measure whether there is a significant difference in survival between the low-expression and the high-expression groups, OncoLnc uses the logrank test. For all but one (*CISH* in LIHC) gene-cohort pairs that had a significant FDR corrected p value in the Cox regression, the logrank p values were also significant (p < 0.05), indicating that these genes are indeed predictive for survival.

### Inferring a pan-cancer survival network

Even though the pan-cancer approach reduces substantially the number of genes used for survival prediction (74%) as compared to the single-cohort approach, with the most important features having a high biological plausibility, there are still 12,082 features that are selected as potentially important in at least one of the 100 replications of the pan-cancer training procedure. In each replication, there are a maximum of 500 genes selected for survival prediction (cf. STAR Methods), meaning that among the total 12,082 genes, there are many genes that are only selected in a small number of replications and the selected genes thus still highly depend on the composition of the training set. Furthermore, this still large number of genes makes it difficult to infer mechanistic information and lacks biological focus. On the other hand, these features might still be relevant for a subset of patients, and simply neglecting the features with low feature importance weights with some threshold might be somewhat arbitrary. The resulting distribution of feature importance weights resembles a “long-tail” distribution often visible with cancer-associated SNPs (Armenia et al., 2018) (Figure S3).

**Figure 4:**
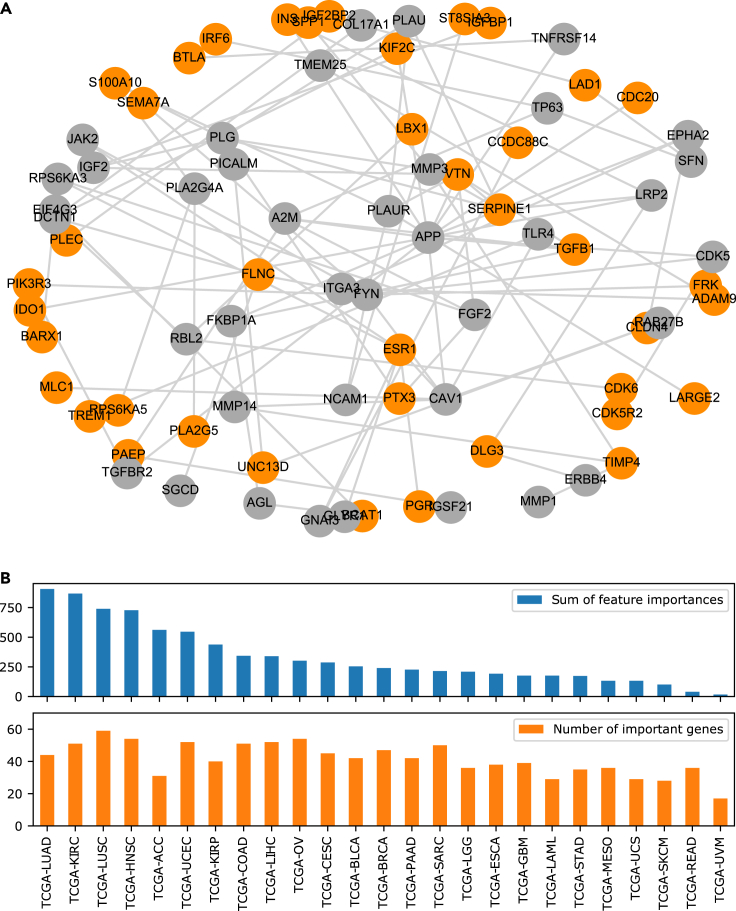
Pan-cancer survival network (A) Largest network module identified by NetCore (Barel and Herwig, 2020) network propagation and module identification based on pan-cancer important features. Orange nodes correspond to seed genes, while genes that were inferred during network propagation are colored in gray. (B) Feature importance of the 103 module genes in single-cohort training (100 replications). Top: Sum of feature importance scores of the module genes per cohort. Bottom: Number of genes (of the 103 module genes) per cohort that are among the important features in single-cohort training (feature importance > 0). See also Tables S2 and S3.

It has been argued that plausibility of machine learning methods could be improved by incorporating prior knowledge on biological networks in the analysis workflow (Camacho et al., 2018), and we challenged this claim by applying our recently developed network propagation method, NetCore, to the feature importance weights of the pan-cancer training approach (cf. STAR Methods). Network propagation has emerged as a useful tool for inferring mechanistic information, in particular from high-throughput data (Cowen et al., 2017). In network propagation genes are organized in a graph and edges between genes are set through biological *a priori* knowledge. Typically, protein-protein interaction networks are used as a scaffold for the interaction context (Huang et al., 2018). For this study, we used the large integrated protein-protein interaction network from the ConsensusPathDB comprising 10,586 nodes and 114,341 interactions (Herwig et al., 2016). Gene nodes were initially weighted with the sums of the feature importance scores over 100 replications of the pan-cancer training and the NetCore propagation method was used to spread these weights over the network until a steady-state condition was reached (Barel and Herwig, 2020). Then we inferred a pan-cancer survival network by applying NetCore’s module identification approach to the network propagation results with the top 100 genes that are contained in the protein-protein interaction network and have the largest feature importance sums as seed genes. NetCore extends these seed genes by genes that were assigned a significantly high weight after the network propagation step to form connected subgraphs, which are called modules (cf. STAR Methods). Figure 4A shows the largest network module identified by NetCore. Overall, 13 network modules containing between 2 and 79 genes were identified. These modules comprised 103 genes in total, 76 genes with high pan-cancer feature importance and 27 additionally inferred genes added in the network propagation steps. The genes and their initial as well as propagated weights are listed in Table S2.

The 103 genes are informative on all different cancer subtypes with an average of 41.48 genes gaining feature importance for the cohorts in single-cohort training. TCGA-LUSC (59 genes) shares the largest number of genes followed by TCGA-HNSC (54) and TCGA-OV (54) (Figure 4B). On the level of feature importance weights, however, the difference is more drastic. While for many cohorts the contributions are high, for TCGA-MESO, TCGA-UCS, TCGA-SKCM, TCGA-READ and TCGA-UVM, the feature importance weights of the 103 genes are rather low. This might in part due to the fact that these cohorts were in the lower range of patient numbers that contributed to pan-cancer training (<200; Table S1).

The pan-cancer survival network has an overlap of 27 genes with previously annotated cancer genes (Repana et al., 2019). Interestingly, the majority of these cancer genes (16 out of 27) were inferred by network propagation, meaning that they had very little to no feature importance with respect to survival prognosis but were re-ranked significantly because of their high connectivity in the protein-protein interaction network (Table S2). The largest re-ranked pan-cancer feature importance is attributed to the gene *PIK3R3* (Phosphoinositide-3-Kinase Regulatory Subunit 3) (Table S2). *PIK3R3* is an enzyme that participates in multiple cancer-related signaling pathways, most importantly PI3K-AKT-mTOR signaling. It can act as an oncogene in colorectal cancer and high expression of *PIK3R3* inhibits cell senescence and promotes cell proliferation (Chen et al., 2020).

### Pan-cancer survival network is strongly associated with the tumor microenvironment

In order to characterize the genes of the pan-cancer survival network we performed over-representation analysis and interrogated canonical pathways and upstream regulators using QIAGEN Ingenuity Pathway Analysis (IPA) (Krämer et al., 2014) (Table 1).

**Table 1:** Over-represented pathways (p < 0.001) computed with QIAGEN Ingenuity Pathway Analysis (IPA)

Ingenuity canonical pathway	-log(p value)	Ratio	Molecules
Tumor microenvironment pathway	9.34	6.25 × 10^−2^	*FGF2, IDO1, IGF2, JAK2, MMP1, MMP14, MMP3, PIK3R3, PLAU, SPP1, TGFB1*
Glucocorticoid receptor signaling	8.60	3.25 × 10^−2^	*A2M, CAV1, ESR1, JAK2, MMP1, MMP3, PGR, PIK3R3, PLA2G4A, PLA2G5, PLAU, RPS6KA5, SERPINE1, TGFB1, TGFBR2*
Role of tissue factor in cancer	8.55	7.76 × 10^−2^	*FRK, FYN, ITGA3, JAK2, MMP1, PIK3R3, PLAUR, RPS6KA3, RPS6KA5*
Hepatic fibrosis signaling pathway	6.85	3.17 × 10^−2^	*FGF2, GNAI3, INS, ITGA3, JAK2, MMP1, PIK3R3, SERPINE1, SPP1, TGFB1, TGFBR2, TLR4*
Hepatic fibrosis/Hepatic stellate cell activation	6.77	4.84 × 10^−2^	*A2M, COL17A1, FGF2, IGF2, MMP1, SERPINE1, TGFB1, TGFBR2, TLR4*
Coagulation system	6.31	1.43 × 10^−1^	*A2M, PLAU, PLAUR, PLG, SERPINE1*
HOTAIR regulatory pathway	6.18	5.00 × 10^−2^	*ESR1, MMP1, MMP14, MMP3, PIK3R3, SPP1, TGFB1, TLR4*
Osteoarthritis pathway	6.15	4.09 × 10^−2^	*DKK1, FGF2, ITGA3, MMP1, MMP3, SPP1, TGFB1, TGFBR2, TLR4*
Growth hormone signaling	6.08	8.45 × 10^−2^	*A2M, IGF2, JAK2, PIK3R3, RPS6KA3, RPS6KA5*
Inhibition of matrix metalloproteases	6.06	1.28 × 10^−1^	*A2M, MMP1, MMP14, MMP3, TIMP4*
Glioma invasiveness signaling	6.01	8.22 × 10^−2^	*PIK3R3, PLAU, PLAUR, PLG, TIMP4, VTN*
Reelin signaling in neurons	5.87	5.74 × 10^−2^	*APP, ARHGEF3, CDK5, FRK, FYN, ITGA3, PIK3R3*
Axonal Guidance signaling	5.62	2.43 × 10^−2^	*ADAM9, CDK5, DPYSL5, EPHA2, FYN, GNAI3, ITGA3, MMP1, MMP14, MMP3, PIK3R3, SEMA7A*
Estrogen receptor signaling	5.62	3.05 × 10^−2^	*CAV1, ESR1, GNAI3, IGF2, JAK2, MMP1, MMP14, MMP3, PGR, PIK3R3*
Leukocyte extravasation signaling	5.57	4.15 × 10^−2^	*CLDN4, GNAI3, ITGA3, MMP1, MMP14, MMP3, PIK3R3, TIMP4*
HIF1A signaling	5.37	3.90 × 10^−2^	*FGF2, IGF2, MMP1, MMP14, MMP3, PIK3R3, SERPINE1, TGFB1*
Semaphorin signaling in neurons	5.12	8.33 × 10^−2^	*CDK5, DPYSL3, DPYSL5, FYN, SEMA7A*
Neuroinflammation signaling pathway	5.05	3.00 × 10^−2^	*APP, JAK2, MMP3, PIK3R3, PLA2G4A, PLA2G5, TGFB1, TGFBR2, TLR4*
Molecular mechanisms of cancer	4.87	2.50 × 10^−2^	*ARHGEF3, CDK5, CDK6, FYN, GNAI3, ITGA3, JAK2, PIK3R3, TGFB1, TGFBR2*
Tec kinase signaling	4.86	4.05 × 10^−2^	*FRK, FYN, GNAI3, ITGA3, JAK2, PIK3R3, TLR4*
p38 MAPK signaling	4.80	5.08 × 10^−2^	*PLA2G4A, PLA2G5, RPS6KA3, RPS6KA5, TGFB1, TGFBR2*
Colorectal cancer metastasis signaling	4.71	3.16 × 10^−2^	*JAK2, MMP1, MMP14, MMP3, PIK3R3, TGFB1, TGFBR2, TLR4*
Caveolar-mediated endocytosis signaling	4.70	6.85 × 10^−2^	*CAV1, FLNC, FYN, INS, ITGA3*
Atherosclerosis signaling	4.61	4.72 × 10^−2^	*MMP1, MMP3, PLA2G4A, PLA2G5, TGFB1, TNFRSF14*
ERK/MAPK signaling	4.43	3.47 × 10^−2^	*ESR1, FYN, ITGA3, PIK3R3, PLA2G4A, PLA2G5, RPS6KA5*
Semaphorin neuronal repulsive signaling pathway	4.39	4.32 × 10^−2^	*CDK5, DPYSL3, DPYSL5, FYN, ITGA3, PIK3R3*
Oncostatin M signaling	4.37	9.30 × 10^−2^	*JAK2, MMP1, MMP3, PLAU*
Role of osteoblasts, osteoclasts and Chondrocytes in rheumatoid arthritis	4.22	3.21 × 10^−2^	*DKK1, MMP1, MMP14, MMP3, PIK3R3, SPP1, TGFB1*
Sperm motility	4.16	3.14 × 10^−2^	*EPHA2, ERBB4, FRK, FYN, JAK2, PLA2G4A, PLA2G5*
Bladder cancer signaling	4.10	5.15 × 10^−2^	*FGF2, MMP1, MMP14, MMP3, RPS6KA5*
Cardiac hypertrophy signaling (enhanced)	4.07	2.01 × 10^−2^	*ADRA1A, ADRA1D, FGF2, GNAI3, ITGA3, JAK2, PIK3R3, RPS6KA5, TGFB1, TGFBR2*
Role of macrophages, fibroblasts and endothelial cells in rheumatoid arthritis	4.05	2.55 × 10^−2^	*DKK1, FGF2, JAK2, MMP1, MMP3, PIK3R3, TGFB1, TLR4*
Chronic myeloid leukemia signaling	3.98	4.85 × 10^−2^	*CDK6, PIK3R3, RBL2, TGFB1, TGFBR2*
Insulin secretion signaling pathway	3.91	2.87 × 10^−2^	*EIF4G3, FYN, INS, JAK2, PCSK2, PIK3R3, RPS6KA5*
CNTF signaling	3.89	7.02 × 10^−2^	*JAK2, PIK3R3, RPS6KA3, RPS6KA5*
T cell exhaustion signaling pathway	3.84	3.43 × 10^−2^	*BTLA, JAK2, PIK3R3, TGFB1, TGFBR2, TNFRSF14*
Regulation of the epithelial mesenchymal transition by growth factors pathway	3.67	3.19 × 10^−2^	*FGF2, JAK2, MMP1, PIK3R3, TGFB1, TGFBR2*
RhoGDI signaling	3.66	3.17 × 10^−2^	*ARHGEF3, CDH10, CDH6, ESR1, GNAI3, ITGA3*
IL-15 production	3.65	4.13 × 10^−2^	*EPHA2, ERBB4, FRK, FYN, JAK2*
Agranulocyte adhesion and diapedesis	3.61	3.11 × 10^−2^	*CLDN4, GNAI3, ITGA3, MMP1, MMP14, MMP3*
Senescence pathway	3.60	2.55 × 10^−2^	*CDK6, PIK3R3, RBL2, RPS6KA5, SERPINE1, TGFB1, TGFBR2*
Role of MAPK signaling in inhibiting the pathogenesis of influenza	3.43	5.33 × 10^−2^	*PLA2G4A, PLA2G5, RPS6KA3, TLR4*
mTOR signaling	3.41	2.86 × 10^−2^	*EIF4G3, FKBP1A, INS, PIK3R3, RPS6KA3, RPS6KA5*
Inhibition of angiogenesis by TSP1	3.32	8.82 × 10^−2^	*FYN, TGFB1, TGFBR2*
MIF-mediated glucocorticoid regulation	3.32	8.82 × 10^−2^	*PLA2G4A, PLA2G5, TLR4*
Necroptosis signaling pathway	3.13	3.18 × 10^−2^	*FKBP1A, PLA2G4A, PLA2G5, RBL2, TLR4*
Cardiac hypertrophy signaling	3.11	2.50 × 10^−2^	*ADRA1A, ADRA1D, GNAI3, PIK3R3, TGFB1, TGFBR2*
MIF regulation of innate immunity	3.04	7.14 × 10^−2^	*PLA2G4A, PLA2G5, TLR4*

Pathway,annotated pathway name; -log(p value),-log of enrichment p value computed with Fisher's exact test; ratio,proportion of genes in the network module that map to the respective pathway and overall number of genes in the pathway; molecules, network module genes that overlap with the pathway.

The genes in the survival network are most strongly enriched with genes from the TME (p = 4.57E-10; *FGF2*, *IDO1*, *IGF2*, *JAK2*, *MMP1*, *MMP14*, *MMP3*, *PIK3R3*, *PLAU*, *SPP1*, *TGFB1*; Table 1). The TME is composed of a variety of host cells, secreted factors as well as extracellular matrix proteins that are strongly interacting with the cancer cells, and this interaction is critical for tumor progression and influences the survival of the patient. The TME is built of several specialized microenvironments such as hypoxic niche, immune microenvironment, and metabolism microenvironment (Jin and Jin, 2020). Several of these specialized TME microenvironments are enriched by the pan-cancer survival network, for example HIF1A signaling (p = 4.27 × 10^−6^; *FGF2*, *IGF2*, *MMP1*, *MMP14*, *MMP3*, *PIK3R3*, *SERPINE1*, *TGFB1*). Hypoxia is correlated with cancer progression. It is a potent factor that influences the characteristics of tumor and stromal cells to support metastasis and *HIF-1* and *HIF-2* genes are associated with poor patient survival (Rankin et al., 2016). Hypoxia leads to the upregulation of *VEGF* and other growth factors such as *FGF2* and, thus, stimulates angiogenesis (De Palma et al., 2017).

Additionally, the pan-cancer survival network holds strong immune-related enrichment signals. For example, glucocorticoid receptor (GR) signaling has been found enriched by the survival network (p = 2.51 × 10^−9^; *A2M*, *CAV*1, *ESR1*, *JAK2*, *MMP1*, *MMP3*, *PGR*, *PIK3R3*, *PLA2G4A*, *PLA2G5*, *PLAU*, *RPS6KA5*, *SERPINE1*, *TGFB1*, *TGFBR2*). Increasing GR signaling in the TME has been recently associated with dysfunctional CD8+ tumor-infiltrating lymphocytes (TILs), which might interfere with the positive effect of TILs on the survival (Acharya et al., 2020). Such positive effects have been shown for example in colon cancer (Idos et al., 2020) and subtypes of breast cancer (Denkert et al., 2018). Further immune cell phenotypes relate to dysfunctions of the immune system and major mechanisms by which tumors escape immunosurveillance. One such phenotype is T cell exhaustion (p = 1.44 × 10^−4^; *BTLA*, *JAK2*, *PIK3R3*, *TGFB1*, *TGFBR2*, *TNFRSF14*). T cell exhaustion manifests in decreased effector cytokine production and impaired cytotoxicity and determines a chronic infectious state which is prone to cancer immune evasion (Jiang et al., 2015).

The pan-cancer survival network further enriches the inhibition of matrix metalloproteases (*A2M*, *MMP1*, *MMP14*, *MMP3*, *TIMP4*). Matrix metalloproteinases (MMPs) are among the most prominent family of proteinases associated with tumorigenesis. They show increased expression in cancer patients and are potent regulators of the TME and other signaling pathways related to cell growth, inflammation and angiogenesis (Kessenbrock et al., 2010).

**Figure 5:**
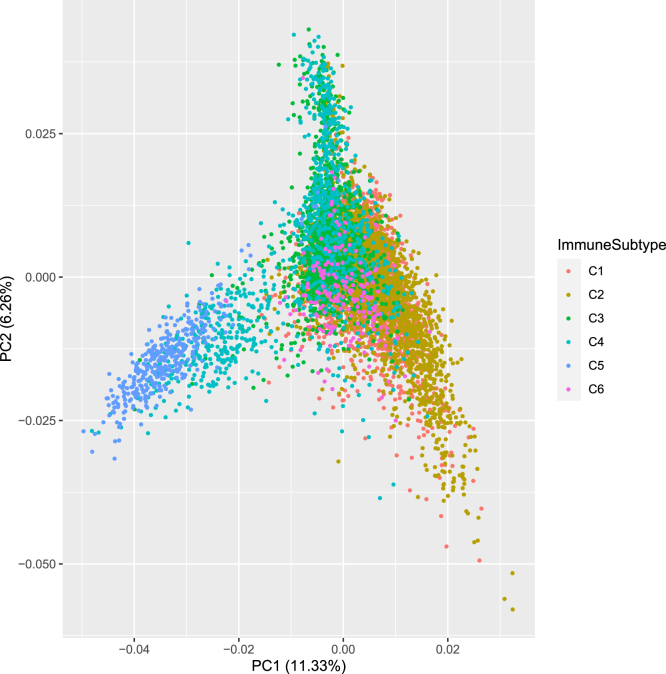
Association of the pan-cancer survival network with immune subtypes. Principal component analysis (PCA) of the patients that can be assigned to an immune subtype according to (Thorsson et al., 2018). The PCA is based on the 103 module genes and patients are colored by their assigned immune subtype. PCA is generated with the R library ggplot2 (Wickham, 2009).

The strong involvement of the TME raises the question to what extent the gene expression signals of the 103 genes are indicative of the immune status of the patients. We explored a recent work that classified the TCGA patients into six immune subtypes (Thorsson et al., 2018). For 7,475 of the 8,024 patients used here the immune subtype could be assigned and principal component analysis of these patients with respect to the gene expression matrix of the 103 genes shows a partial distinction of these immune subtypes (Figure 5). In particular, immune subtype C5 (immunologically quiet), that consisted mostly of brain lower-grade gliomas (LGG), could be separated from the other groups as well as parts of the patients of subtype 2 (IFN-γ dominant). This shows that the gene expression of the pan-cancer survival network genes holds information on the immune status of the patients.

We were further interested in potential regulators of the pan-cancer survival network that are frequently mutated in cancer samples. We therefore performed an enrichment analysis with the annotation sets of “upstream regulators” in the IPA system and found a total of 47 significantly enriched upstream regulators (p value < 1 × 10^−5^) that had been identified as cancer driver or potential cancer driver genes in a recent cancer gene annotation effort (Repana et al., 2019). The top 15 cancer-relevant upstream regulators are (Table S3): *JUN* (20 target genes in the network), *TNF* (34), *IL1B* (25), *TP53* (33), *IL1A* (13), *FGF2* (15), *MAP3K1* (7), *EGFR* (15), *STAT3* (16), *HRAS* (16), *CDH1* (7), *AKT1* (11), *PTEN* (16), *FOXO1* (13), and *SMARCA4* (15). Interestingly, several of these factors have been associated with the TME. For example, *TNF* (*tumor necrosis factor alpha*) is a factor in the regulation of the TME. It is a multifunctional key cytokine in apoptosis and cell survival as well as in inflammation and immunity (van Horssen et al., 2006). *STAT3* (*signal transducer and activator of transcription 3*) is key in regulating the anti-tumor immune response and a therapeutic target of immune therapies in different cancer subtypes such as celecoxib in colorectal cancer and pyrimethamine in chronic lymphocytic leukemia (Zou et al., 2020). *TP53* is among the most studied genes in cancer and has numerous roles in particular in escaping apoptosis. Additionally, it has been shown that *TP53* can act non-cell autonomously to suppress tumorigenesis by promoting an antitumor microenvironment, in part through secreted factors that modulate macrophage function (Lujambio et al., 2013).

Thus, we conclude that the pan-cancer survival network emphasizes the role of the TME for the immune status and the survival prognosis of cancer patients.

## Discussion

Cancer survival is a multi-factorial problem and has important implications for patient care and therapy choices. The purpose of this work was to derive a prediction model for cancer survival with high biological plausibility. We achieved this by combining an ensemble tree approach based on gradient boosting along with network propagation with a comprehensive protein-protein interaction network.

In the first part of this work, we applied XGBoost tree ensemble learning to gene expression data to predict patient survival in 25 different cancer types. To overcome the observable shortcomings of single-cohort training such as low number of training samples and intra-cohort heterogeneity of patients, and to empower the identification of cross-cohort features, we next applied pan-cancer training and showed improved performance of the method, although the number of features used is reduced by 74%. In order to reduce the number of features further and to gain biological plausibility of the prediction model we revealed a pan-cancer network of 103 genes with our recent network propagation algorithm, NetCore, utilizing a high-confidence protein-protein interaction network. The computed network module is predominantly indicative of the TME as was shown by enrichment analysis and correlation to the immune status of the patients. The role of the TME for cancer progression and metastases as well as for the response to therapies has been emphasized (Quail and Joyce, 2013), and our findings particularly highlight the importance of the hypoxic and immune-related parts of the TME. Additionally, we identified a decent negative correlation (R = −0.55) of the methods performances with the age distribution of the patients. This may also suggest that the aging TME influences cancer progression and survival (Fane and Weeraratna, 2020), and that this aging TME is hard to resolve by machine learning approaches, while signatures appear more predictive in younger, and presumably more intact, states. Survival prognosis can benefit from these findings by taking into account age-specific training and validation cohorts.

Besides the TME- and immune-related pathways, the pan-cancer survival network enriches further signaling pathways that have high cross-cohort relevance for survival such as mTOR-signaling. The mechanistic target of rapamycin (mTOR) pathway regulates fundamental cell processes and dysregulation of the pathway is relevant for the progression of cancer (Saxton and Sabatini, 2017). In our network mTOR signaling (p = 2.86 × 10^−2^; *EIF4G3*, *FKBP1A*, *INS*, *PIK3R3*, *RPS6KA3*, *RPS6KA5*) is activated by PI3K/AKT in response to insulin (*INS*) (Zoncu et al., 2011). *INS* and *PI3KR3* were among the top 100 genes judged by the feature importance weights of the pan-cancer XGBoost prediction model and were used as seed genes for the network propagation. Another very prominent pathway in cancer is ERK/MAPK signaling (p = 3.47 × 10^−2^; *ESR1*, *FYN*, *ITGA3*, *PIK3R3*, *PLA2G4A*, *PLA2G5*, *RPS6KA5*), which is the core of the signaling network involved in regulating cell growth, development, and division and a target of many cancer therapeutics. Interestingly, associated proteins highlight the cooperation with the estrogen receptor 1 (*ESR1*). It has been reported that the cooperation of the estrogen and MAPK signaling is due to *ESR1-ERK2* cooperative binding and enhances the proliferative effect of estrogen signaling (Madak-Erdogan et al., 2011). *ESR1* is a frequently mutated cancer gene, in particular in metastatic breast cancer (Razavi et al., 2018), and was identified among the top 100 important features by the XGBoost model.

We based our survival prediction method on gene expression data of cancer patients from multiple cancer types. Gene expression data has been found to be the most informative omics datatype in different biomedical prediction tasks (Costello et al., 2014; Vale Silva and Rohr, 2020). In principle, the XGBoost survival prediction approach described in this work can easily be extended by additional clinical and molecular data types like DNA methylation, mutation, or copy number variation (CNV) data and such a multi-omics approach has recently been applied to pan-cancer survival prediction using deep learning (Cheerla and Gevaert, 2019). These additional data types could complement the information contained in gene expression data, but on the other hand, incorporating additional data and, thus, also additional model features, while the number of patients does not increase, would exacerbate the curse of dimensionality (Bellman, 2015; Keogh and Mueen, 2017), a phenomenon regularly encountered in machine learning that is often responsible for model overfitting.

There are different strategies to counteract this phenomenon, including the application of dimensionality reduction techniques such as principal component analysis (PCA) (Hotelling, 1933; Pearson, 1901) to the data before feeding it to the machine learning algorithm, or the incorporation of feature selection steps into the learning procedure. Feature selection techniques for supervised learning problems can be divided into three categories, namely filter methods, wrapper methods, and embedded methods (Saeys et al., 2007). Filter methods select features by considering intrinsic properties of the data like correlation between features or the chi-square test. However, they ignore the relation between the features and the target variable. In contrast, wrapper methods do consider dependencies between features and target variable by evaluating different feature subsets with the machine learning method used for the prediction task and selecting the subset of features that yields the best model performance. A disadvantage of this type of feature selection is however that it is computationally intensive, especially for datasets with many features, because the number of potential feature subsets that need to be tested grows exponentially with the number of features. The third type of feature selection methods, the embedded techniques, also consider relations between the features and the target variable by using the selected machine learning method to evaluate features, but instead of evaluating model performance for different sets of features, they use built-in feature importance measures of the machine learning method and are thus computationally far less intensive than wrapper methods. In our XGBoost-based cancer survival prediction method, we chose to integrate an embedded feature selection approach, which uses 4-fold cross-validation and applies simple XGBoost models to the training data in order to reduce the number of features used in each model replication from 60,483 to 500.

When combining the results of the survival prediction model with network propagation, we are able to explore the functional content of the genes identified as important features by XGBoost. As a measure of feature importance, we chose ‘gain’ over other possible feature importance measures such as ‘weight’ and ‘cover’ (see https://xgboost.readthedocs.io/en/latest/python/python_api.html), because ‘gain’ measures the relative importance of a feature for the prediction result with respect to the prediction improvement that this feature brings. In contrast, ‘weight’ simply counts how often a feature is used to split the data in any of the regression trees and does not consider where in the tree the corresponding split is used. This is relevant because splits closer to the root of the tree are typically more important for the prediction result than splits further down, where only few samples are affected by the split (an example of a regression tree from the pan-cancer XGBoost model can be found in Figure S4). The feature importance measure ‘cover’ on the other hand does take into account where in a tree the split is made; however, it does so by only considering the number of samples affected by the splits this feature is used for, and does not consider how often the feature is used or how it affects the prediction result. Thus, we deemed ‘gain’ the most appropriate feature importance measure for the purpose of identifying genes relevant for survival.

We showed improved survival prediction performance when using pan-cancer instead of single-cohort training. On the one hand, it is likely that a considerable proportion of this improvement can be attributed to the much larger number of training patients. This major advantage for machine learning has been shown recently in other application domains, for example, drug sensitivity testing (Lloyd et al., 2021). In fact, when performing the pan-cancer XGBoost training procedure (including feature selection and hyperparameter tuning) on randomly sampled subsets of different sizes, it becomes apparent that for many of the cancer types the performance drops with smaller training samples (Figure S5). On the other hand, however, we observe that pan-cancer features are generalizable whereas single-cohort features are very specific to the respective cancer type. Interestingly, the type of features is also changing drastically. While with single-cohort training 40.4% of the important features refer to protein coding genes, this number increases to 56.5% in pan-cancer training. Accordingly, the proportion of the other feature types (lncRNAs and processed pseudogenes) drops considerably. This reflects the high level of specificity of lncRNAs and pseudogenes.

To the best of our knowledge this is the first study that applies XGBoost to pan-cancer survival prognosis. Pan-cancer survival prediction has recently also been addressed by deep learning methods like VAECox (Kim et al., 2020) and MultiSurv (Vale-Silva and Rohr, 2021). VAECox pre-trains a variational autoencoder on pan-cancer gene expression data and then transfers the learned weights to a survival prediction neural network architecture, which is then fine-tuned separately on each cancer cohort. MultiSurv uses multimodal data including omics data as well as clinical information and imaging data to predict the conditional survival probabilities for a predefined set of follow-up time intervals. While both methods successfully integrate pan-cancer molecular data to predict cancer survival and report good results, biological interpretation of MultiSurv is limited to visualizing the learned feature representations with t-distributed stochastic neighbor embedding (t-SNE) and no gene- or pathway-level explanations are available. For VAECox, the nodes with the highest variance are extracted from the hidden layers of the model and the Pearson correlations between these nodes and the expression of the gene features are analyzed. While this approach provides biological interpretation to some extent, there are a large number of genes whose expression is correlated with the most variable hidden nodes at different levels, and pathway enrichment analysis conducted directly on these genes might not be able to fully discover the underlying survival mechanisms.

In contrast to these methods, our approach applies network propagation using the NetCore tool to extract biological plausibility of the learned feature representations, which is a fairly generic step that could be applied to any machine learning approach. NetCore performs random walk with restart propagation on protein-protein interactions with a node measure that is different from the node degree and thus is more robust against the inherent node degree bias of PPIs (Barel and Herwig, 2020). This leads to an emphasis on so-called influential nodes that are typically in the core of the network rather than its periphery and thus is highly suited to infer common subnetworks from otherwise highly dispersed nodes. It uses a semi-supervised module identification step by connecting seed nodes of interest with the nodes that become significantly up-weighted after the propagation step. Seed nodes can be identified either by literature knowledge (supervised) or by top experimental signals (unsupervised). Since we wanted to interrogate the ability of the machine learning approach to generate plausible features, we used the unsupervised approach in this study where seed nodes were chosen by the top 100 feature importance weights from the pan-cancer training. Alternative approaches could, for example, incorporate seed nodes derived from existing signatures.

Network propagation has been combined with machine learning in other application domains, for example, drug sensitivity prediction (Manica et al., 2019). This approach leverages the information conferred by protein-protein interactions *a priori* to machine learning to reduce the feature space to genes associated with known drug targets of the evaluated drugs. Additionally, reweighted random survival forest (RRSF) (Wang and Liu, 2018) integrates network propagation in order to incorporate *a priori* information on gene interactions into the learning procedure. Instead of using the network propagation results in a feature selection step where only genes are kept that have a high weight after network propagation, RRSF directly incorporates the weights into model training. This is done by splitting the nodes in the tree growing process of the random forest according to their weight after network propagation, where topologically important genes in the gene interaction network receive higher weights and are thus selected with a higher probability in the node splitting procedure. In contrast to these methods, our approach separates the two analysis components and applies network propagation *a posteriori* to enhance biological plausibility, while leaving the machine learning part unchanged in order to explore all available features and to gain maximal prediction accuracy.

In summary, we have introduced a machine learning approach to pan-cancer survival prediction, which combines ensemble tree boosting with network propagation and highlights the role of the aging TME for survival prognosis.

### Limitations of the study

Our method is based on cancer patient gene expression data provided by the TCGA consortium. Although TCGA comprises a variety of cancer patients from diverse cancer types, and we have implemented rigorous train-test splits and also tested our method on cancer types withheld in model training, we have not evaluated the method on any cancer patient data completely unrelated to the TCGA database.

Furthermore, we considered only one molecular data modality, namely RNA-seq gene expression data, for model training and identification of the pan-cancer survival network. Incorporation of additional molecular data modalities like methylation and mutations could add more information to the model and complement the gene expression data. However, this would come at the cost of introducing more features into the machine learning method, which could increase the risk of overfitting.

## STAR★Methods

### Key resources table

**Table:** 

REAGENT or RESOURCE	SOURCE	IDENTIFIER
**Deposited data**		

HTSeq-FPKM gene expression and clinical files	The Cancer Genome Atlas (TCGA)	https://portal.gdc.cancer.gov/ GDC data releases v22.0 and v24.0
ConsensusPathDB (CPDB) protein-protein interaction network (version 34)	Herwig et al. (2016)	https://github.molgen.mpg.de/barel/NetCore/blob/6860331ae9ff8725e666d936e9b853ef28893a00/data/CPDB_high_confidence.txt
Known and candidate cancer genes from the network of cancer genes (NCG)	Repana et al. (2019)	http://ncg.kcl.ac.uk/index.php NCG version 6.0

**Software and algorithms**		

NetCore	Barel and Herwig (2020)	https://github.molgen.mpg.de/barel/NetCore
OncoLnc	Anaya (2016)	http://www.oncolnc.org/
Single-cohort and pan-cancer XGBoost survival prediction	This Paper	https://github.molgen.mpg.de/thedinga/xgb_survival_network
R Implementations of Path2Surv (MKL[H] and MKL[P]), random survival forest (RF), and survival support vector machine (SVM)	Dereli et al. (2019)	https://github.com/mehmetgonen/path2surv
XGBoost python package	Chen and Guestrin (2016)	https://github.com/dmlc/xgboost/tree/master/python-package
MyGene python package	Xin et al. (2016); Wu et al. (2013)	http://mygene.info version 3.1
QIAGEN ingenuity pathway analysis (QIAGEN IPA)	Krämer et al., 2014	https://digitalinsights.qiagen.com/products-overview/discovery-insights-portfolio/analysis-and-visualization/qiagen-ipa/

### Resource availability

#### Lead contact

Further information and requests for resources should be directed to and will be fulfilled by the lead contact, Ralf Herwig (herwig@molgen.mpg.de).

#### Materials availability

This study did not generate new unique reagents.

### Method details

#### Data and preprocessing

This study is based on gene expression data from The Cancer Genome Atlas (TCGA) consortium (https://www.cancer.gov/tcga), which provides genomic and clinical data for more than 10,000 cancer patients and 33 cancer types through the Genomic Data Commons (GDC) data portal (https://portal.gdc.cancer.gov/). We used gene expression data from primary tumors of these 33 cancer cohorts. We chose to only use samples with sample type annotations "Primary Tumor" and "Primary Blood Derived Cancer - Peripheral Blood" and excluded samples derived from normal tissue and metastatic tumors because these sample types are expected to have different gene expression characteristics than primary tumors. For each cancer type, we downloaded all HTSeq-FPKM files, which contain FPKM-normalized gene expression data, and the corresponding clinical files to extract patient survival data. For the cohorts TCGA-COAD, TCGA-LAML, TCGA-LUAD, and TCGA-LUSC, all data was obtained from GDC data release v22.0 (released January 16, 2020) and for the 29 remaining cohorts, all files were retrieved from GDC data release v24.0 (released May 7, 2020). For training the survival prediction model and comparing performance between methods, we only used cohorts with at least 20 uncensored patients (25 cohorts) since splitting cohorts with less uncensored patients into training and test data and using 20% of patients for testing would have led to only one to four test patients per cohort, thus limiting the informative value of predictions on these cohorts. The remaining eight cohorts with less than 20 uncensored patients (TCGA-CHOL, TCGA-DLBC, TCGA-KICH, TCGA-PCPG, TCGA-PRAD, TCGA-TGCT, TCGA-THCA, and TCGA-THYM) were not part of the training procedure and could thus be used to demonstrate the transferability of the pan-cancer XGBoost survival prediction model to cancer types not seen in training. We furthermore excluded all patients for whom either gene expression measurements or key clinical data, namely age, gender, vital status, and time to either death or censoring, were unavailable or inconsistent. This led to a total of 8,024 patients in the 25 cancer cohorts used for single-cohort and pan-cancer XGBoost training and an additional 1,571 patients in the eight cohorts used for prediction only.

#### The XGBoost method

XGBoost (Chen and Guestrin, 2016) is a supervised machine learning method based on tree ensembles. It implements gradient tree boosting, which predicts the output by additive functions represented by regression trees. More formally, the predicted output ${\hat{y}}_{i}\in R$ for a sample i and its m features $x_{i}\in R^{m}$ is computed as:

$${\hat{y}}_{i}=\varphi \left(x_{i}\right)=\sum_{k=1}^{K}f_{k}\left(x_{i}\right),\;f_{k}\in F,$$

where F is the space of regression trees and K is the number of functions used to predict the output. Each of the trees $f_{k}$ is associated with a set of continuous leaf weights *w* and maps each sample to one of the tree’s leaf indices and its corresponding weight *w*_*i*_. The final prediction for a sample is then made by summing up the leaf weights assigned to the sample by the *K* different trees. In order to learn the tree functions that map samples to leaves, the following objective is minimized:

$$L\left(\varphi \right)=\sum_{i}l\left({\hat{y}}_{i},y_{i}\right)+\sum_{k}\Omega \left(f_{k}\right),$$

where$\Omega \left(f\right)=\gamma T+\frac{1}{2}\lambda w^{2}$ is a regularization term that penalizes complexity of the tree functions to reduce overfitting. Here, *T* is the number of leaves in the tree, w represents the leaf weights and *γ* and λ are model hyperparameters. In the first term of the model objective, *l* is a differentiable convex loss function measuring the difference between the predicted value ${\hat{y}}_{i}$ and the true value *Y*_*i*_. For our task of survival prediction, the loss function is selected to be the negative partial loglikelihood for Cox proportional hazards regression (*objective = survival:cox* in the XGBoost model parameters). This loss function is defined as:

$$l_{Cox}\left(\text{β}\right)=-\sum_{i:E_{i}=1}\left(x_{i}\text{β}-\log\sum_{j:T_{j}\geq T_{i}}e^{x_{j}\text{β}}\right),$$

where $E_{i}$ is a censoring flag with $E_{i}=1$ indicating that patient i is dead (i.e., uncensored) and $E_{i}=0$ indicating that the patient’s survival time is censored, $T_{i}$ is the survival time of patient i, $x_{i}$ are the features of this patient and β is a vector of unknown variables that need to be learned by the model (Cox, 1972). Intuitively, this loss is calculated by comparing each of the uncensored patients to all patients that survived at least as long as this patient in terms of predicted death risk or hazard.

#### Single-cohort and pan-cancer survival prediction with XGBoost

In this study, we applied XGBoost with Cox proportional hazards regression as a learning objective to predict the survival of cancer patients from 25 different cancer types. A graphical overview of how survival risk scores were predicted based on gene expression data can be found in Figure S6.

To assess the performance of the single-cohort and pan-cancer approaches, we trained and evaluated both versions of the method for 100 replications on gene expression datasets from 25 TCGA cohorts. In each replication, the gene expression data was randomly split into 80% training data and 20% test data in a stratified manner, such that training and test sets always contained approximately the same percentage of censored and uncensored patients, and in the pan-cancer approach 80% of each cohort were assigned to the training data and 20% to the test data. Then, the XGBoost-based learning procedure was performed on the training data as described below and the prediction performance was evaluated on the test data by computing the C-Index of the predicted risk scores.

Feature selection: In the first step, a patient×gene expression matrix was constructed, containing the gene expression values from patients either belonging to the same TCGA cohort (single-cohort approach) or to multiple different cohorts (pan-cancer approach). Patients with missing or inconsistent data for age, gender, or survival/censoring time were excluded from the data. Then, an XGBoost-based feature selection procedure was performed to reduce the 60,483 genes measured in TCGA to 500 genes meaningful for survival. In order to identify genes that are relevant to survival not only for a specific training set composition, a stratified 4-fold cross-validation was performed on the training data to find genes that are relevant to survival across all folds. Stratified 4-fold cross-validation in this context means that each of the four data splits contained approximately equal proportions of censored and uncensored patients to ensure comparability across folds. In each step of the cross-validation procedure, we first removed genes with zero mean absolute deviation (MAD) across the three data splits used as training data in this fold. By removing genes with MAD=0, we excluded genes without variability from further feature selection because these genes hold no information value for patient survival. Next, we used XGBoost to identify predictive features for each fold. To this end, we trained 20 XGBoost models with different sets of model hyperparameters and limited size for each fold of the cross-validation in order to identify features that do not depend on the yet untuned model hyperparameters but are relevant to survival on a more general scale. Limited size in this context means that we allowed the XGBoost models to grow a maximum of 5 to 20 regression trees with maximal tree depths between 1 and 3 in order to avoid overfitting and allow the models to select only the most informative features. From each of the trained small XGBoost models, we then extracted the feature importance scores of all genes used as features by the respective model and for each gene calculated the average feature importance across all four rounds of cross-validation and all 20 models per round. As a measure of feature importance we selected ‘gain’, which is defined as the average gain the respective feature brings to the evaluation metric across all decision tree splits in which it is used and thus measures the relative importance of this feature for the prediction result (https://xgboost.readthedocs.io/en/latest/python/python_api.html). In the last step, we then selected the 500 genes with the highest average feature importance scores across all models in the 4-fold cross-validation as features.

Hyperparameter tuning: We performed another 4-fold cross-validation on the training data in each model replication to tune the XGBoost model hyperparameters including maximum tree depth, number of boosting trees, and regularization parameters. We first generated 500 sets of random hyperparameter combinations. In each round of the cross-validation we then trained an XGBoost model for each of these hyperparameter sets on the three training data splits of this round. The training data splits each contained gene expression measurements for the 500 genes identified in the feature selection procedure. The performance of each model was then evaluated on the remaining data split of the round by computing the concordance index (C-Index) of the prediction. The formula for computing the C-Index was adapted from Dereli et al. (2019) and is defined as:

$$\text{C}-\text{Index}=\frac{\sum_{i=1}^{N}\sum_{j\neq i}\Delta_{ij}1\left(\left(y_{i}-y_{j}\right)\left({\hat{y}}_{j}-{\hat{y}}_{i}\right)>0\right)}{\sum_{i=1}^{N}\sum_{j\neq i}\Delta_{ij}},$$

with${\text{Δ}}_{ij}=1$ if patients i and j are comparable, meaning that either both patients are uncensored or patient i is uncensored and patient j is censored, but patient i has a shorter survival time than patient j. Otherwise, if patients i and j are not comparable, ${\text{Δ}}_{ij}=0$. N is the total number of patients for which survival has been predicted, $y_{i}$ is the true survival time of patient i, ${\hat{y}}_{i}$ is the patients predicted risk or hazard score, and the indicator function 1(·) evaluates to 1 whenever its argument is true and 0 otherwise. Intuitively, the C-Index measures the fraction of patient pairs with concordant survival times – meaning the patient with the shorter true survival time is predicted to have a higher death risk – among all comparable pairs of patients. Thus, a larger value of the C-Index indicates a better prediction performance of the evaluated model, while for random predictions the C-Index would be expected to assume a value of 0.5. To select the optimal hyperparameters in each model replication, the average C-Index across all four rounds of the 4-fold cross-validation was computed for each of the 500 tested parameter combinations and the combination of hyperparameters was selected that yielded the best average C-Index. At last, we trained the final XGBoost model on all training patients of the respective model replication, using the gene expression values of the genes identified in the feature selection step and the model hyperparameters selected in the hyperparameter tuning step.

Survival prediction validation: Finally, in each of the 100 model replications, we applied the fully trained model to the 20% left-out patients and computed the C-Index on these patients in order to retrieve patient survival risk scores.

The whole survival prediction procedure, including feature selection, hyperparameter tuning and the final training of the XGBoost survival prediction model, was implemented in Python (release 3.7) and is based on the Python XGBoost package (https://github.com/dmlc/xgboost/tree/master/python-package). The code is available in the following GitHub repository: https://github.molgen.mpg.de/thedinga/xgb_survival_network. The runtime for one model replication of the pan-cancer XGBoost approach – including feature selection and hyperparameter optimization – on all patients from 25 TCGA cancer cohorts, split into 80% training and 20% test data, was approximately 16 hours (on a Linux machine with 64 cores).

#### Other survival prediction methods

We compared our single-cohort XGBoost method against the survival prediction methods random survival forest (Ishwaran et al., 2008), survival support vector machine (Khan and Zubek, 2008; Shivaswamy et al., 2007), and the multiple-kernel learning method Path2Surv (Dereli et al., 2019). Path2Surv is an extension of survival support vector machines that– instead of a single kernel function like in traditional support vector machines – combines multiple kernels in a weighted sum to learn a survival function. Each of these kernels is calculated on expression values of genes forming a pathway or gene set and only informative kernels are considered in the sum by assigning them non-zero weights. This way, Path2Surv uses less gene expression features than for instance survival support vector machines and offers a possibility to assess which pathways or gene sets were relevant for predicting survival. In their publication, Dereli et al. tested Path2Surv for two different gene/pathway sets, namely the Hallmark gene sets (Liberzon et al., 2015) and the Pathway Interaction Database (PID) (Schaefer et al., 2009). We adopted these two versions of Path2Surv to compare against our XGBoost-based survival prediction method. For random survival forest (RF), survival support vector machine (SVM) and Path2Surv, R implementations from Dereli et al. were adapted and the R Optimization Infrastructure (ROI) (Theuβl et al., 2020) was used to solve the quadratic optimization problems in the SVM and Path2Surv methods. While all methods were supplied with all 60,483 gene expression features measured in TCGA and prior to training RF and SVM only genes with zero standard deviation were excluded, Path2Surv additionally excluded features from kernels it considered as uninformative. In each replication of training, the regularization parameter C was tuned for SVM and Path2Surv (range between 1 × 10^−4^ and 1 × 10^5^), and the number of trees to grow was tuned for RF (range between 500 and 2,500 trees) performing a 4-fold cross-validation on the training data, as described in (Dereli et al., 2019). All other parameters were kept as default. For further details we refer the reader to (Dereli et al., 2019).

#### Entropy measurement

Entropy is a measure of uncertainty or information content (Shannon, 1948) and is defined as:

$$H\left(X\right)=-\sum_{i=1}^{n}P\left(x_{i}\right){\log\;}_{2}\left(P\left(x_{i}\right)\right),$$

where$x_{1},\;\ldots ,\;x_{n}$ are the possible outcomes of a random variable X and $P\left(x_{i}\right)$ is the probability of $x_{i}$. If all outcomes $x_{1},\;\ldots ,\;x_{n}$ are equally likely, the entropy will be maximal, while the entropy is minimal in the scenario where only one of the possible outcomes occurs with absolute certainty. We transferred this concept to the single-cohort feature importance weights, which were computed by building the sum of feature importance scores over the 100 model replications of the XGBoost model for each cohort, to measure how well a gene generalizes as a feature over different cancer types. To this end, we computed a probability matrix from the single-cohort feature importance weights by dividing each weight score by the sum of scores for this gene over all 25 cohorts and used this probability matrix to compute the entropies of all genes. If a gene was identified as an important feature across all 25 TCGA cohorts with similar feature importance weights it has high entropy, while a gene that is only among the predictive features in one cancer cohort has minimal entropy of zero. Thus, genes with a high entropy are equally important for predicting survival with the single-cohort XGBoost method in different cancer types and are identified to generalize well, while genes with a low entropy are likely to be cancer-type specific.

#### Inference of a pan-cancer survival network with NetCore

We used network propagation to infer a pan-cancer survival network from the important features identified in the 100 replications of the pan-cancer XGBoost approach. For each gene from the pan-cancer important features, we calculated a feature importance weight as the sum of feature importance scores over all 100 replications, where the feature had a positive feature importance score if it was among the important features of this replications and a score of 0 if it was not used for survival prediction by XGBoost in this replication. Ensembl Gene Identifiers were converted to Hugo Symbols using the MyGene Python package (version 3.1, http://mygene.info) (Wu et al., 2013; Xin et al., 2016) and gene entities that did not map to a Hugo Symbol were removed. All genes together with their feature importance weights were then fed to NetCore (Barel and Herwig, 2020). NetCore is a random walk with restart network propagation method that uses node coreness instead of node degree for normalization in order to address the node degree bias of protein-protein interaction networks (PPIs). Following the network propagation procedure, NetCore also provides a module identification approach, where phenotype-associated network modules are identified in a semi-supervised manner. These network modules are sub-networks of the PPI and comprise so called seed genes – in our case the top 100 genes from the important features that are included in the PPI and have the highest feature importance weights – as well as additional genes identified in the network propagation step that function as links between seed genes. For the cancer survival module identification, NetCore was applied on a high confidence protein-protein interaction network from ConsensusPathDB (Herwig et al., 2016), which was obtained from the NetCore GitHub repository (https://github.molgen.mpg.de/barel/NetCore) and comprises 10,586 genes and 114,341 interactions. For the random walk with restart, the default restart probability of 0.8 was chosen.

#### Over-representation analysis

Over-representation analysis (ORA) measures the overlap between a set of genes (i.e., the most important survival features) and another pre-defined gene set called functional gene set. We used QUIAGEN's IPA software (Krämer et al., 2014) to derive ORA results with respect to canonical pathway and upstream regulator target sets. The ORA was judged with Fisher’s exact test.

### Quantification and statistical analysis

#### Comparison of model performances

To compare the performance of the single-cohort XGBoost method with the RF, SVM, and Path2Surv methods and the pan-cancer with the single-cohort XGBoost method, we trained each method for 100 replications and in each replication computed the concordance index (C-Index). The mean C-Indices of the compared methods were then analyzed with the Wilcoxon unpaired rank-sum test, as implemented in the R function stat_compare_means, which is part of the ggpubr library (https://www.rdocumentation.org/packages/ggpubr). We selected a significance threshold of 0.05 for the Wilcoxon unpaired rank-sum test and adopted the method’s default values for symbols indicating further significance levels (ns: p>0.05, ∗:p≤0.05, ∗∗:p≤0.01, ∗∗∗:p≤0.001, ∗∗∗∗:p≤0.0001).

#### Significance thresholds

Throughout the entire manuscript, we consider a pvalue below 0.05 as significant.

## Data Availability

• The data used in this study is publicly available through the Genomic Data Commons (GDC) data portal (https://portal.gdc.cancer.gov/). • The code generated during this study is available at https://github.molgen.mpg.de/thedinga/xgb_survival_network. • All other resources are listed in the Key resources table.

## Footnotes

Supplemental information can be found online at https://doi.org/10.1016/j.isci.2021.103617.

## References

[bib1] Acharya N., Madi A., Zhang H., Klapholz M., Escobar G., Dulberg S., Christian E., Ferreira M., Dixon K.O., Fell G. (2020). Endogenous glucocorticoid signaling regulates CD8+ T cell differentiation and development of dysfunction in the tumor microenvironment. Immunity.

[bib2] Alexandrov L.B., Kim J., Haradhvala N.J., Huang M.N., Tian Ng A.W., Wu Y., Boot A., Covington K.R., Gordenin D.A., Bergstrom E.N. (2020). The repertoire of mutational signatures in human cancer. Nature.

[bib3] Allemani C., Matsuda T., Di Carlo V., Harewood R., Matz M., Nikšić M., Bonaventure A., Valkov M., Johnson C.J., Estève J. (2018). Global surveillance of trends in cancer survival 2000–14 (CONCORD-3): analysis of individual records for 37 513 025 patients diagnosed with one of 18 cancers from 322 population-based registries in 71 countries. Lancet.

[bib4] Allemani C., Weir H.K., Carreira H., Harewood R., Spika D., Wang X.-S., Bannon F., Ahn J.V., Johnson C.J., Bonaventure A. (2015). Global surveillance of cancer survival 1995–2009: analysis of individual data for 25 676 887 patients from 279 population-based registries in 67 countries (CONCORD-2). Lancet.

[bib5] Anaya J. (2016). OncoLnc: linking TCGA survival data to mRNAs, miRNAs, and lncRNAs. PeerJ Comput.Sci..

[bib6] Armenia J., Wankowicz S.A.M., Liu D., Gao J., Kundra R., Reznik E., Chatila W.K., Chakravarty D., Han G.C., Coleman I. (2018). The long tail of oncogenic drivers in prostate cancer. Nat. Genet..

[bib7] Aunan J.R., Cho W.C., Søreide K. (2017). The biology of aging and cancer: a brief overview of shared and divergent molecular hallmarks. Aging Dis..

[bib8] Barel G., Herwig R. (2020). NetCore: a network propagation approach using node coreness. Nucleic Acids Res..

[bib9] Bellman R.E. (2015).

[bib10] Camacho D.M., Collins K.M., Powers R.K., Costello J.C., Collins J.J. (2018). Next-generation machine learning for biological networks. Cell.

[bib11] Cheerla A., Gevaert O. (2019). Deep learning with multimodal representation for pancancer prognosis prediction. Bioinformatics.

[bib12] Cheetham S.W., Faulkner G.J., Dinger M.E. (2020). Overcoming challenges and dogmas to understand the functions of pseudogenes. Nat. Rev. Genet..

[bib13] Chen Q., Sun X., Luo X., Wang J., Hu J., Feng Y. (2020). PIK3R3 inhibits cell senescence through p53/p21 signaling. Cell Death Dis..

[bib14] Chen T., Guestrin C. (2016). Proceedings of the 22nd ACM SIGKDD International Conference on Knowledge Discovery and Data Mining. Presented at the KDD ’16: The 22nd ACM SIGKDD International Conference on Knowledge Discovery and Data Mining, ACM, San Francisco California USA.

[bib15] Ching T., Zhu X., Garmire L.X. (2018). Cox-nnet: an artificial neural network method for prognosis prediction of high-throughput omics data. PLoS Comput.Biol..

[bib16] Costello J.C., Heiser L.M., Georgii E., Gönen M., Menden M.P., Wang N.J., Bansal M., Ammad-ud-din M., Hintsanen P., Khan S.A. (2014). A community effort to assess and improve drug sensitivity prediction algorithms. Nat. Biotechnol..

[bib17] Cowen L., Ideker T., Raphael B.J., Sharan R. (2017). Network propagation: a universal amplifier of genetic associations. Nat. Rev. Genet..

[bib18] Cox D.R. (1972). Regression models and life-tables. J. R. Stat. Soc. SeriesB Methodol..

[bib19] De Palma M., Biziato D., Petrova T.V. (2017). Microenvironmental regulation of tumour angiogenesis. Nat. Rev. Cancer.

[bib20] Denkert C., von Minckwitz G., Darb-Esfahani S., Lederer B., Heppner B.I., Weber K.E., Budczies J., Huober J., Klauschen F., Furlanetto J. (2018). Tumour-infiltrating lymphocytes and prognosis in different subtypes of breast cancer: a pooled analysis of 3771 patients treated with neoadjuvant therapy. Lancet Oncol..

[bib21] Dereli O., Oğuz C., Gönen M. (2019). Path2Surv: pathway/gene set-based survival analysis using multiple kernel learning. Bioinformatics.

[bib22] Fane M., Weeraratna A.T. (2020). How the ageing microenvironment influences tumour progression. Nat. Rev. Cancer.

[bib23] Gao G.F., Parker J.S., Reynolds S.M., Silva T.C., Wang L.-B., Zhou W., Akbani R., Bailey M., Balu S., Berman B.P. (2019). Before and after: comparison of legacy and harmonized TCGA genomic data commons’ data. Cell Syst..

[bib24] Herwig R., Hardt C., Lienhard M., Kamburov A. (2016). Analyzing and interpreting genome data at the network level with ConsensusPathDB. Nat. Protoc..

[bib25] Hoadley K.A., Yau C., Hinoue T., Wolf D.M., Lazar A.J., Drill E., Shen R., Taylor A.M., Cherniack A.D., Thorsson V. (2018). Cell-of-Origin patterns dominate the molecular classification of 10,000 tumors from 33 types of cancer. Cell.

[bib26] Hotelling H. (1933). Analysis of a complex of statistical variables into principal components. J. Educ. Psychol..

[bib27] Huang J.K., Carlin D.E., Yu M.K., Zhang W., Kreisberg J.F., Tamayo P., Ideker T. (2018). Systematic evaluation of molecular networks for discovery of disease genes. Cell Syst..

[bib28] Idos G.E., Kwok J., Bonthala N., Kysh L., Gruber S.B., Qu C. (2020). The prognostic implications of tumor infiltrating lymphocytes in colorectal cancer: a systematic review and meta-analysis. Sci. Rep..

[bib29] Ishwaran H., Kogalur U.B., Blackstone E.H., Lauer M.S. (2008). Random survival forests. Ann. Appl. Stat..

[bib30] Jiang Y., Li Y., Zhu B. (2015). T-cell exhaustion in the tumor microenvironment. Cell Death Dis..

[bib31] Jin M.-Z., Jin W.-L. (2020). The updated landscape of tumor microenvironment and drug repurposing. Sig Transduct Target.Ther..

[bib32] Katzman J.L., Shaham U., Cloninger A., Bates J., Jiang T., Kluger Y. (2018). DeepSurv: personalized treatment recommender system using a Cox proportional hazards deep neural network. BMC Med. Res. Methodol..

[bib33] Keogh E., Mueen A., Sammut C., Webb G.I. (2017). Encyclopedia of Machine Learning and Data Mining.

[bib34] Kessenbrock K., Plaks V., Werb Z. (2010). Matrix metalloproteinases: regulators of the tumor microenvironment. Cell.

[bib35] Khan F.M., Zubek V.B. (2008). 2008 Eighth IEEE International Conference on Data Mining. Presented at the 2008 Eighth IEEE International Conference on Data Mining (ICDM), IEEE, Pisa, Italy.

[bib36] Kim S., Kim K., Choe J., Lee I., Kang J. (2020). Improved survival analysis by learning shared genomic information from pan-cancer data. Bioinformatics.

[bib37] Kourou K., Exarchos T.P., Exarchos K.P., Karamouzis M.V., Fotiadis D.I. (2015). Machine learning applications in cancer prognosis and prediction. Comput.Struct.Biotechnol. J..

[bib38] Krämer A., Green J., Pollard J., Tugendreich S. (2014). Causal analysis approaches in ingenuity pathway analysis. Bioinformatics.

[bib39] Leiserson M.D.M., Vandin F., Wu H.-T., Dobson J.R., Eldridge J.V., Thomas J.L., Papoutsaki A., Kim Y., Niu B., McLellan M. (2015). Pan-cancer network analysis identifies combinations of rare somatic mutations across pathways and protein complexes. Nat. Genet..

[bib40] Li Y., Wang J., Ye J., Reddy C.K. (2016). Proceedings of the 22nd ACM SIGKDD International Conference on Knowledge Discovery and Data Mining. Presented at the KDD ’16: The 22nd ACM SIGKDD International Conference on Knowledge Discovery and Data Mining, ACM, San Francisco California USA.

[bib41] Liberzon A., Birger C., Thorvaldsdóttir H., Ghandi M., Mesirov J.P., Tamayo P. (2015). The molecular signatures database hallmark gene set collection. Cell Syst..

[bib42] Liu J., Lichtenberg T., Hoadley K.A., Poisson L.M., Lazar A.J., Cherniack A.D., Kovatich A.J., Benz C.C., Levine D.A., Lee A.V. (2018). An integrated TCGA pan-cancer clinical data resource to drive high-Quality survival outcome analytics. Cell.

[bib43] Lloyd J.P., Soellner M.B., Merajver S.D., Li J.Z. (2021). Impact of between-tissue differences on pan-cancer predictions of drug sensitivity. PLoS Comput. Biol..

[bib44] Lochhead P., Imamura Y., Morikawa T., Kuchiba A., Yamauchi M., Liao X., Qian Z.R., Nishihara R., Wu K., Meyerhardt J.A. (2012). Insulin-like growth factor 2 messenger RNA binding protein 3 (IGF2BP3) is a marker of unfavourable prognosis in colorectal cancer. Eur. J. Cancer.

[bib45] Lujambio A., Akkari L., Simon J., Grace D., Tschaharganeh D.F., Bolden J.E., Zhao Z., Thapar V., Joyce J.A., Krizhanovsky V. (2013). Non-cell-autonomous tumor suppression by p53. Cell.

[bib46] Madak-Erdogan Z., Lupien M., Stossi F., Brown M., Katzenellenbogen B.S. (2011). Genomic collaboration of estrogen receptor α and extracellular signal-regulated kinase 2 in regulating gene and proliferation programs. Mol. Cell Biol..

[bib47] Mancarella C., Scotlandi K. (2020). IGF2BP3 from physiology to cancer: novel discoveries, Unsolved issues, and future perspectives. Front. Cell Dev. Biol..

[bib48] Manica M., Oskooei A., Born J., Subramanian V., Sáez-Rodríguez J., Rodríguez Martínez M. (2019). Toward explainable anticancer compound sensitivity prediction via multimodal attention-based convolutional encoders. Mol. Pharmaceutics.

[bib49] Murdoch W.J., Singh C., Kumbier K., Abbasi-Asl R., Yu B. (2019). Definitions, methods, and applications in interpretable machine learning. Proc. Natl. Acad. Sci. U S A.

[bib50] Ogunleye A., Wang Q.-G. (2020). XGBoost model for chronic kidney disease diagnosis. IEEE/ACM Trans. Comput. Biol. Bioinf..

[bib51] Parris T.Z. (2020). Pan-cancer analyses of human nuclear receptors reveal transcriptome diversity and prognostic value across cancer types. Sci. Rep..

[bib52] Patil P., Bachant-Winner P.-O., Haibe-Kains B., Leek J.T. (2015). Test set bias affects reproducibility of gene signatures. Bioinformatics.

[bib53] Pearson K. (1901). LIII. *On lines and planes of closest fit to systems of points in space*. Lond.Edinb.Dublin Philosophical. Mag. J. Sci..

[bib54] Quail D.F., Joyce J.A. (2013). Microenvironmental regulation of tumor progression and metastasis. Nat. Med..

[bib55] Rankin E.B., Nam J.-M., Giaccia A.J. (2016). Hypoxia: signaling the metastatic cascade. Trends Cancer.

[bib56] Razavi P., Chang M.T., Xu G., Bandlamudi C., Ross D.S., Vasan N., Cai Y., Bielski C.M., Donoghue M.T.A., Jonsson P. (2018). The genomic landscape of endocrine-resistant advanced breast cancers. Cancer Cell.

[bib57] Repana D., Nulsen J., Dressler L., Bortolomeazzi M., Venkata S.K., Tourna A., Yakovleva A., Palmieri T., Ciccarelli F.D. (2019). The Network of Cancer Genes (NCG): a comprehensive catalogue of known and candidate cancer genes from cancer sequencing screens. Genome Biol..

[bib58] Saeys Y., Inza I., Larranaga P. (2007). A review of feature selection techniques in bioinformatics. Bioinformatics.

[bib59] Sanchez-Vega F., Mina M., Armenia J., Chatila W.K., Luna A., La K.C., Dimitriadoy S., Liu D.L., Kantheti H.S., Saghafinia S. (2018). Oncogenic signaling pathways in the cancer genome Atlas. Cell.

[bib60] Saxton R.A., Sabatini D.M. (2017). mTOR signaling in growth, metabolism, and disease. Cell.

[bib61] Schaefer C.F., Anthony K., Krupa S., Buchoff J., Day M., Hannay T., Buetow K.H. (2009). PID: the pathway interaction database. Nucleic Acids Res..

[bib62] Shannon C.E. (1948). A mathematical theory of communication. Bell Syst. Tech. J..

[bib63] Shivaswamy P.K., Chu W., Jansche M. (2007). Seventh IEEE International Conference on Data Mining (ICDM 2007). Presented at the Seventh IEEE International Conference on Data Mining (ICDM 2007), IEEE, Omaha, NE, USA.

[bib64] Siegel R.L., Miller K.D., Jemal A. (2018). Cancer statistics, 2018: cancer statistics, 2018. CACancer J. Clin..

[bib65] Statello L., Guo C.-J., Chen L.-L., Huarte M. (2021). Gene regulation by long non-coding RNAs and its biological functions. Nat. Rev. Mol. Cell Biol..

[bib66] The ICGC/TCGA Pan-Cancer Analysis of Whole Genomes Consortium (2020). Pan-cancer analysis of whole genomes. Nature.

[bib67] Theußl S., Schwendinger F., Hornik K. (2020). **Roi** : an extensible *R* optimization infrastructure. J. Stat. Soft..

[bib68] Thorsson V., Gibbs D.L., Brown S.D., Wolf D., Bortone D.S., Ou Yang T.-H., Porta-Pardo E., Gao G.F., Plaisier C.L., Eddy J.A. (2018). The immune landscape of cancer. Immunity.

[bib69] Torlay L., Perrone-Bertolotti M., Thomas E., Baciu M. (2017). Machine learning–XGBoost analysis of language networks to classify patients with epilepsy. Brain Inf..

[bib70] Vale Silva L.A., Rohr K. (2020). 2020 IEEE 17th International Symposium on Biomedical Imaging (ISBI). Presented at the 2020 IEEE 17th International Symposium on Biomedical Imaging (ISBI), IEEE, Iowa City, IA, USA.

[bib71] Vale-Silva L.A., Rohr K. (2021). Long-term cancer survival prediction using multimodal deep learning. Sci. Rep..

[bib72] van Horssen R., ten Hagen T.L.M., Eggermont A.M.M. (2006). TNF-α in cancer treatment: molecular insights, antitumor effects, and clinical utility. Oncologist.

[bib73] Venet D., Dumont J.E., Detours V. (2011). Most random gene expression signatures are significantly associated with breast cancer outcome. PLoS Comput.Biol..

[bib74] Wang W., Liu W. (2018). Integration of gene interaction information into a reweighted random survival forest approach for accurate survival prediction and survival biomarker discovery. Sci. Rep..

[bib75] Wickham H. (2009).

[bib76] Wu C., MacLeod I., Su A.I. (2013). BioGPS and MyGene.info: organizing online, gene-centric information. Nucleic Acids Res..

[bib77] Xin J., Mark A., Afrasiabi C., Tsueng G., Juchler M., Gopal N., Stupp G.S., Putman T.E., Ainscough B.J., Griffith O.L. (2016). High-performance web services for querying gene and variant annotation. Genome Biol..

[bib78] Zoncu R., Efeyan A., Sabatini D.M. (2011). mTOR: from growth signal integration to cancer, diabetes and ageing. Nat. Rev. Mol. Cell Biol..

[bib79] Zou S., Tong Q., Liu B., Huang W., Tian Y., Fu X. (2020). Targeting STAT3 in cancer immunotherapy. Mol. Cancer.

