# Supplementary material for: A gradient tree boosting and network propagation derived pan-cancer survival network of the tumor microenvironment
Source: iScience. 2021 Dec 11;25(1):103617. doi: 10.1016/j.isci.2021.103617 (PMC8786644; doi:10.1016/j.isci.2021.103617)
Supplement: Document S1. Figures S1–S6 and Tables S1–S3 [file mmc1.pdf]

iScience, Volume *25*

Supplemental information

A gradient tree boosting and network propagation derived pan-cancer survival network of the tumor microenvironment

Kristina Thedinga and Ralf Herwig

Supplemental Figures

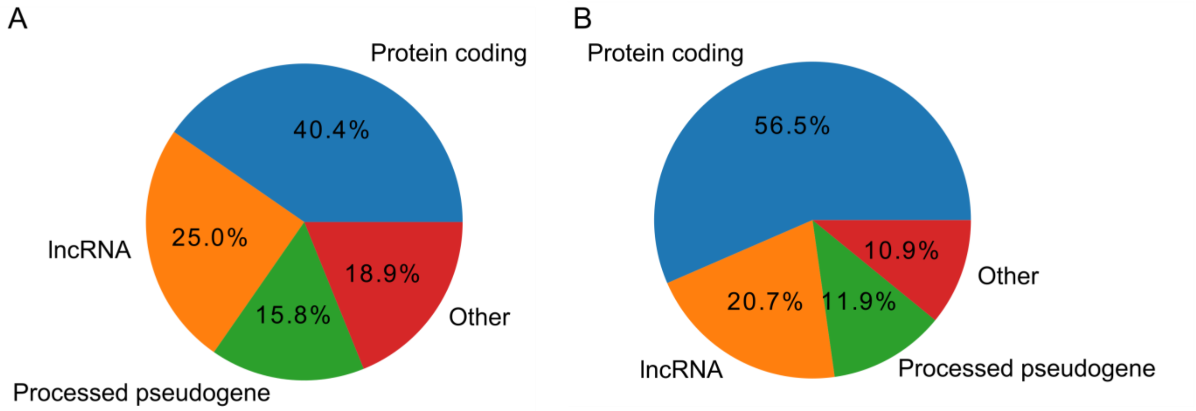

Figure S1. Types of important features identified in single-cohort and pan-cancer training, Related to Figure 2. RNA types were obtained using the MyGene Python package (version 3.1, http://mygene.info) (Wu et al., 2013; Xin et al., 2016). **A** Percentages of different types of RNAs identified as important features in the single-cohort XGBoost approach. **B** Percentages of different types of RNAs identified as important features in the pan-cancer XGBoost approach.

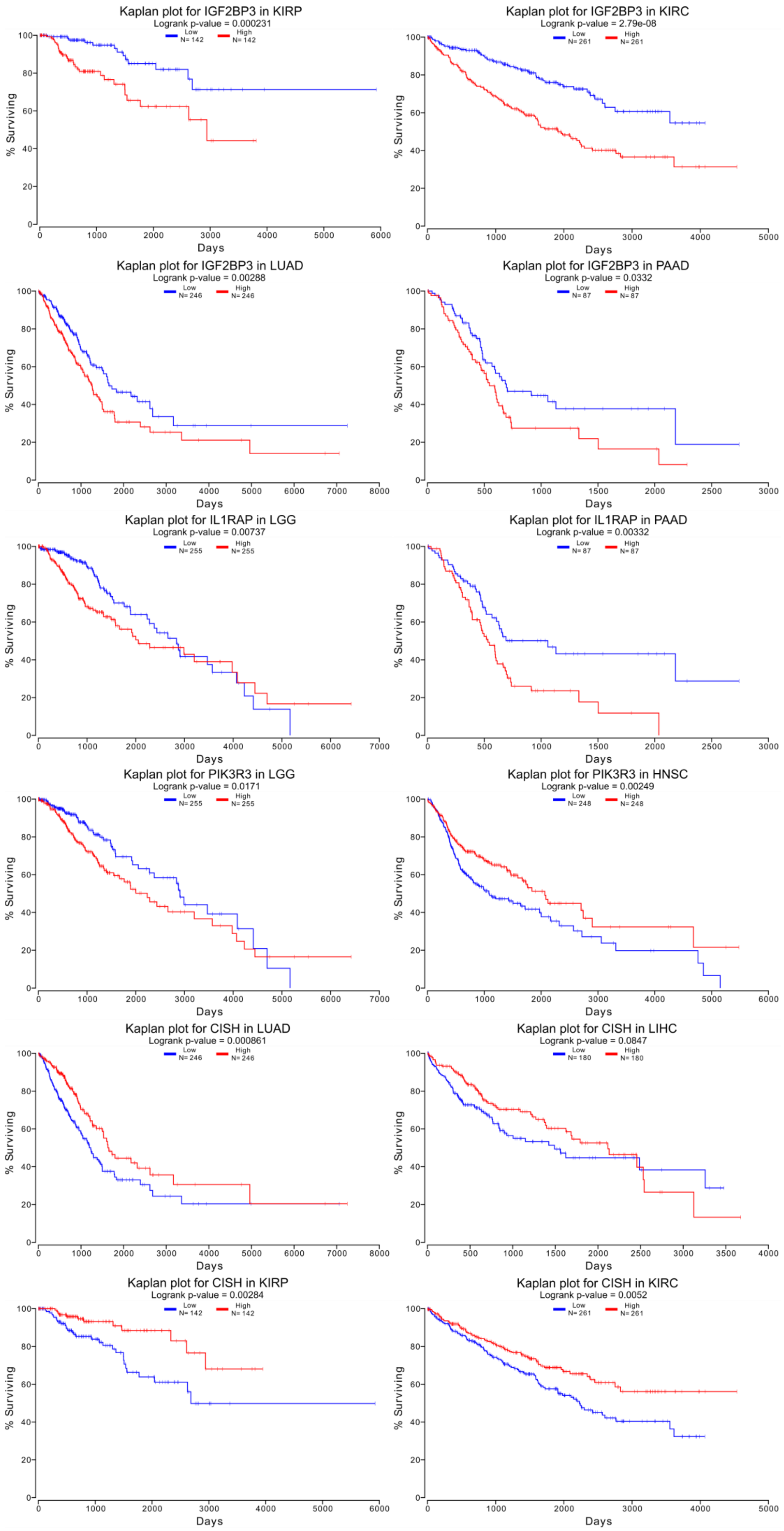
Kaplan plot for IGF2BP3 in KIRP
Logrank p-value = 0.000231
Low N= 142
High N= 142
Kaplan plot for IGF2BP3 in KIRC
Logrank p-value = 2.79e-08
Low N= 261
High N= 261
Kaplan plot for IGF2BP3 in LUAD
Logrank p-value = 0.00288
Low N= 246
High N= 246
Kaplan plot for IGF2BP3 in PAAD
Logrank p-value = 0.0332
Low N= 87
High N= 87
Kaplan plot for IL1RAP in LGG
Logrank p-value = 0.00737
Low N= 255
High N= 255
Kaplan plot for IL1RAP in PAAD
Logrank p-value = 0.00332
Low N= 87
High N= 87
Kaplan plot for PIK3R3 in LGG
Logrank p-value = 0.0171
Low N= 255
High N= 255
Kaplan plot for PIK3R3 in HNSC
Logrank p-value = 0.00249
Low N= 248
High N= 248
Kaplan plot for CISH in LUAD
Logrank p-value = 0.000861
Low N= 246
High N= 246
Kaplan plot for CISH in LIHC
Logrank p-value = 0.0847
Low N= 180
High N= 180
Kaplan plot for CISH in KIRP
Logrank p-value = 0.00284
Low N= 142
High N= 142
Kaplan plot for CISH in KIRC
Logrank p-value = 0.0052
Low N= 261
High N= 261
% Surviving
Days

Figure S2. Additional Kaplan-Meier plots for *IGF2BP3*, *IL1RAP*, *PIK3R3*, and *CISH*, Related to Figure 3. The Kaplan-Meier plots shown here were obtained from OncoLnc (Anaya, 2016) and correspond to the four (KIRP, KIRC, LUAD and PAAD), two (LGG and PAAD), two (LGG and HNSC), and four (LUAD, LIHC, KIRP, and KIRC) additional cohorts that were not shown in Figure 3, but also show significant survival performance (FDR < 0.05 in Cox regression) in the OncoLnc analyses for *IGF2BP3*, *IL1RAP*, *PIK3R3*, and *CISH*, respectively. For grouping the patients into two groups the 50th percentile of gene expression was selected as a cutoff in all cases.

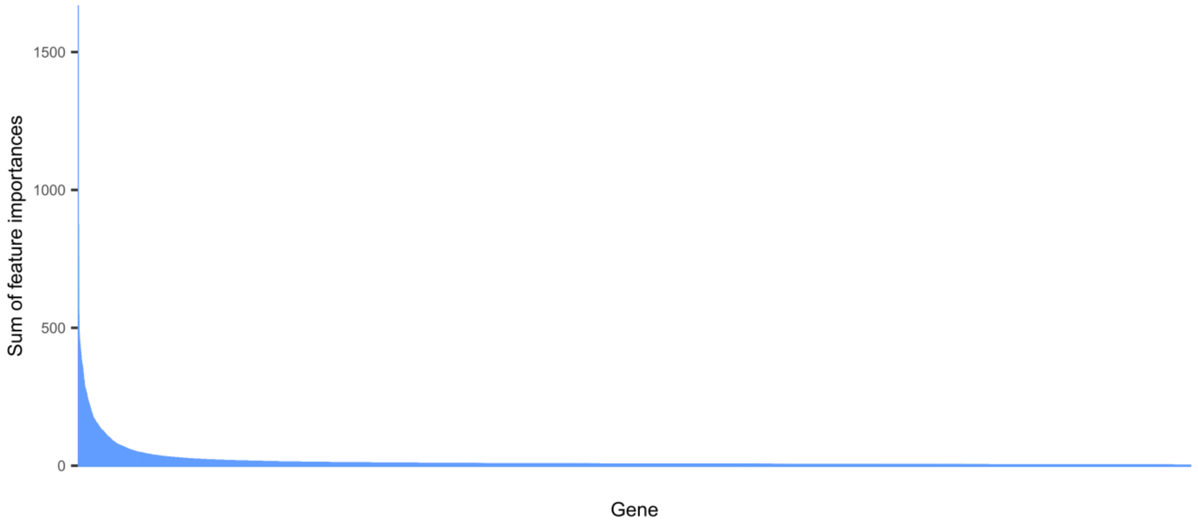

Figure S3. “Long-tail” distribution of the pan-cancer feature importance weights, Related to Figure 3. The x-axis displays the 12,082 genes identified as important features in the 100 model replications of the pan-cancer XGBoost method and the y-axis shows the corresponding sums of feature importance scores across the 100 replications.

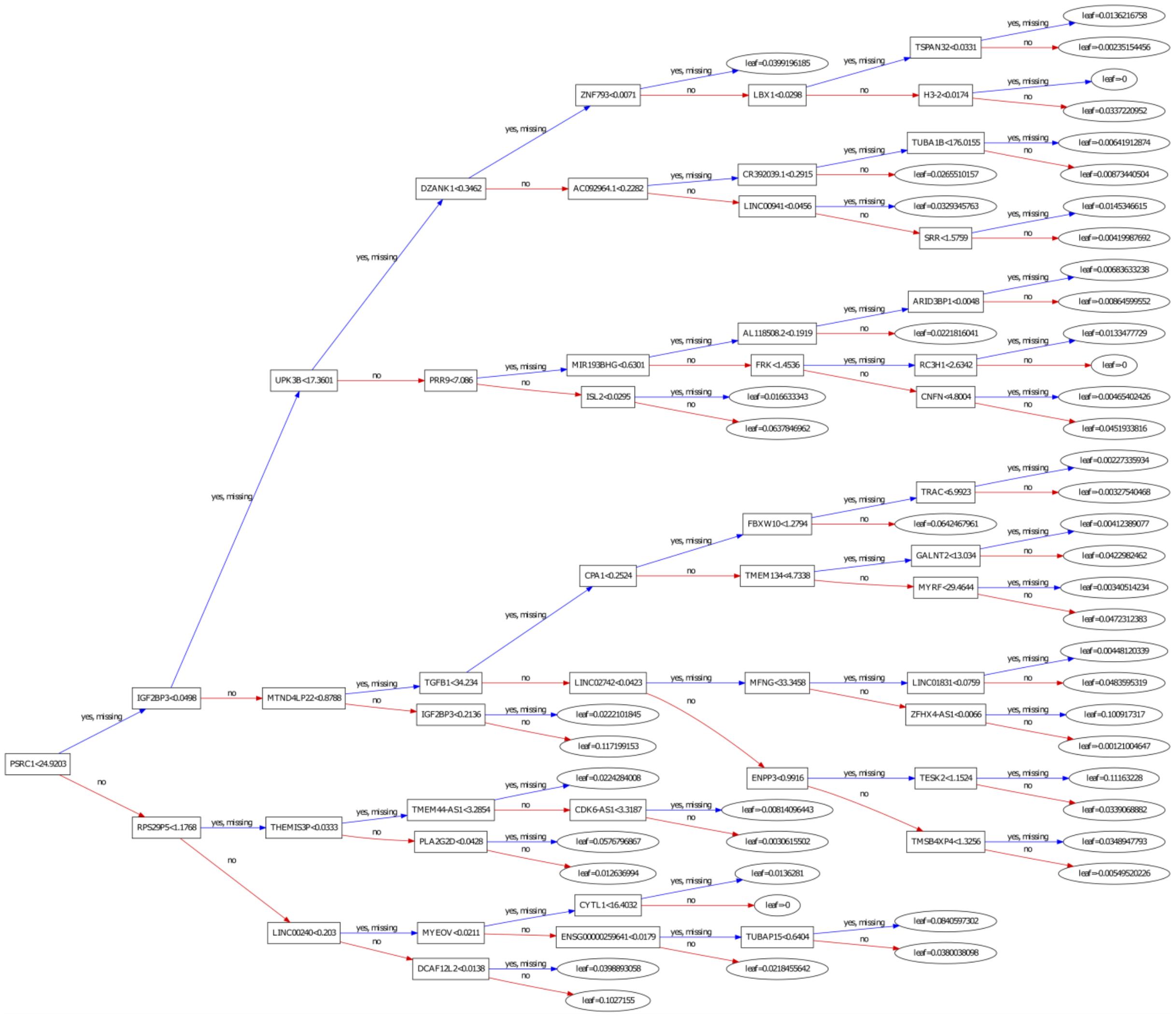

Figure S4. Exemplary regression tree from the pan-cancer XGBoost model, Related to STAR methods. Rectangular nodes represent gene expression thresholds (genes are denoted by their Hugo Symbol obtained with the MyGene Python package (version 3.1, http://mygene.info) (Wu et al., 2013; Xin et al., 2016) if available and otherwise by their Ensembl Identifier) and elliptical leaf nodes represent predictions. Since an XGBoost model consists of multiple regression trees, these predictions do not represent the predicted risk scores of the model. The tree graph was visualized with https://edotor.net/.

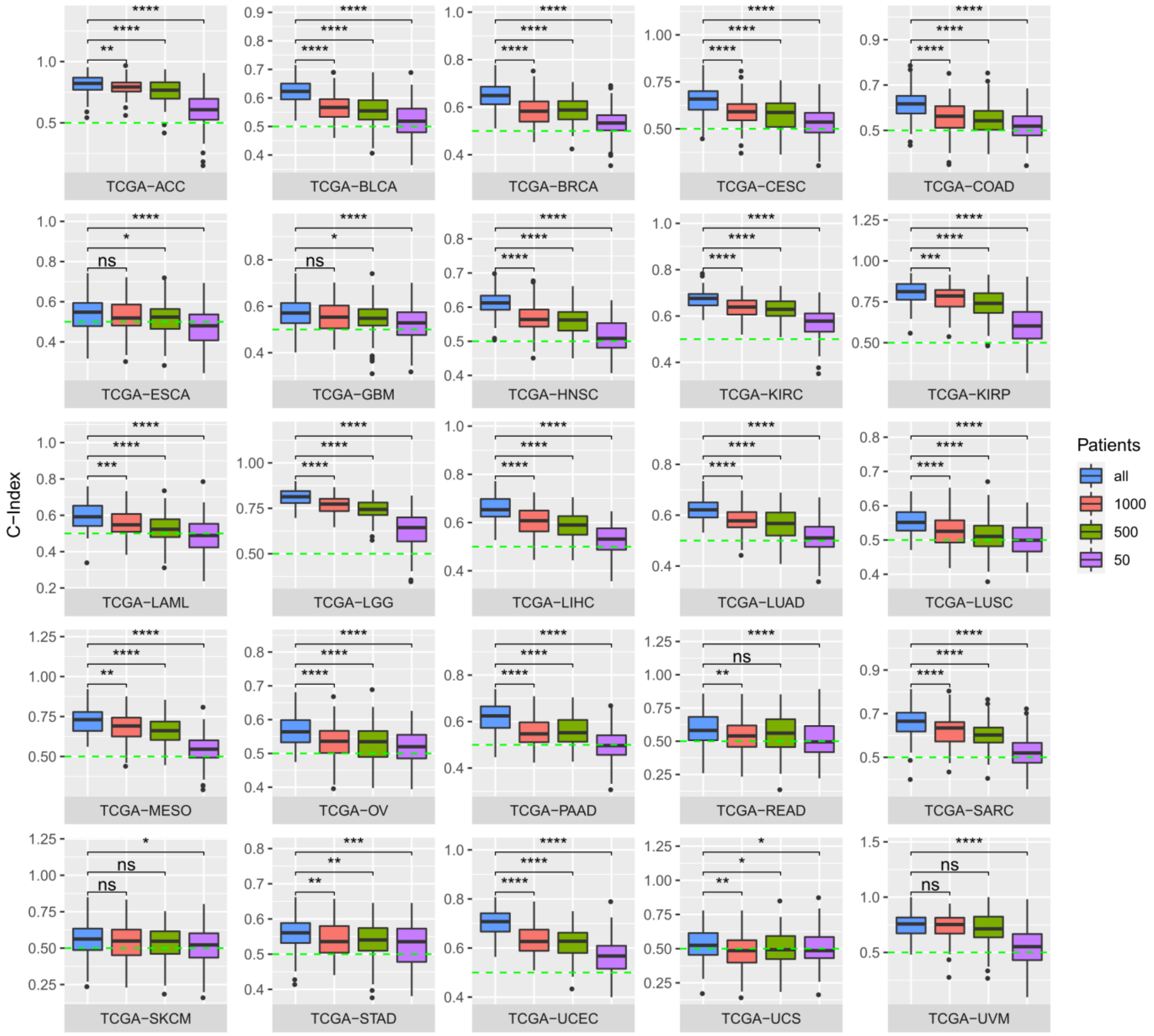

Figure S5. Pan-cancer XGBoost training on random patient subsets, Related to Figure 2. The results obtained from 100 replications of pan-cancer model training are compared between training on all pan-cancer patients (N = 6,419) contained in the training data of the respective replication (blue) and training on patient subsets of different sizes. For training on the patient subsets, in each replication a pre-defined number of patients (either 1,000, 500, or 50) was randomly selected from the training data of the respective replication, where each subset contained approximately the same number of patients from each of the 25 TCGA cohorts. This random patient subsampling was performed prior to the feature selection step, such that feature selection as well as hyperparameter tuning and the training of the final survival prediction model were performed on this patient subset only. Model evaluation was then done on all patients belonging to the test data of the respective replication and no subsampling was performed. Mean C-Indices were compared with the Wilcoxon unpaired rank sum test and significance levels are defined as ns: $p > 0.05$, $* : p \leq 0.05$, $** : p \leq 0.01$, $*** : p \leq 0.001$, $**** : p \leq 0.0001$.

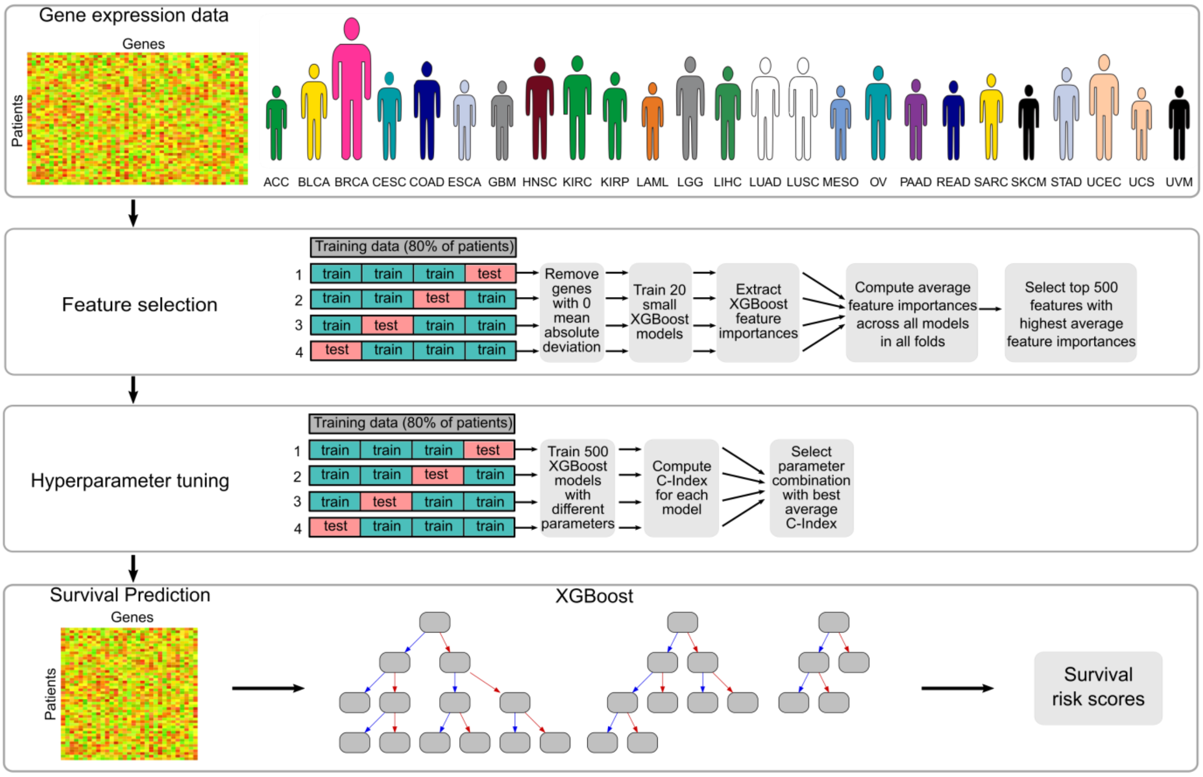

Figure S6. Schematic of the XGBoost training procedure, Related to STAR methods. The XGBoost training procedure is based on a *patient* × *gene expression* matrix comprising patients from either one cancer cohort (single-cohort approach) or multiple cancer cohorts (pan-cancer approach). From this gene expression matrix, a subset of 500 genes is selected in a feature selection step that includes a 4-fold cross-validation of training small XGBoost models on the whole data and selecting the features with the highest average feature importance scores across models. Next, another 4-fold cross-validation is performed to tune the model hyperparameters and finally, a survival prediction model is trained on the reduced gene expression matrix of 500 genes and with the optimized model hyperparameters.

Supplemental Tables

Table S1. Summary of the 33 TCGA cancer cohorts, Related to STAR methods. Number of Pan-Cancer Features refers to the number of pan-cancer features that were also among the important features in single-cohort training for the respective cohort. Information of the organ system of each cancer cohort was obtained from (Parris, 2020). More detailed information on the different cancer types can be found at https://www.cancer.gov/about-nci/organization/ccg/research/structural-genomics/tcga/studied-cancers.

Cohort Abbreviation	Cohort Name	Organ System	Number of Pan-Cancer Features	Number of Patients	Number of Uncen-sored Patients	Median Age (IQR)	Gender
**TCGA-ACC**	Adrenocor-tical carcinoma	Endocrine	2027	79	28	49.0 (24.50)	48 female, 31 male
**TCGA-BLCA**	Bladder urothelial carcinoma	Urologic	4814	401	176	68.0 (16.00)	104 female, 297 male
**TCGA-BRCA**	Breast invasive carcinoma	Gyneco-logic	4864	1068	149	58.0 (18.25)	1056 female, 12 male
**TCGA-CESC**	Cervical squamous cell carcinoma and endocervical adenocar-cinoma	Gyneco-logic	4359	291	72	46.0 (18.00)	291 female
**TCGA-COAD**	Colon adenocar-cinoma	Gastroin-testinal	4719	433	95	68.0 (19.00)	200 female, 233 male
**TCGA-ESCA**	Esophageal carcinoma	Gastroin-testinal	4048	160	63	60.0 (19.00)	23 female, 137 male
**TCGA-GBM**	Glioblas-toma multiforme	Central nervous system	4351	151	122	60.0 (18.00)	54 female, 97 male
**TCGA-HNSC**	Head and neck squamous	Head and neck	5157	498	217	61.0 (16.00)	132 female, 366 male

	cell carcinoma						
**TCGA-KIRC**	Kidney renal clear cell carcinoma	Urologic	4605	526	171	60.0 (17.00)	183 female, 343 male
**TCGA-KIRP**	Kidney renal papillary cell carcinoma	Urologic	3540	283	44	61.0 (17.00)	75 female, 208 male
**TCGA-LAML**	Acute myeloid leukemia	Hemato-logic and lymphatic malignan-cies	3827	130	78	55.5 (24.75)	60 female, 70 male
**TCGA-LGG**	Brain lower grade glioma	Central nervous system	3999	505	125	41.0 (20.00)	226 female, 279 male
**TCGA-LIHC**	Liver hepato-cellular carcinoma	Gastroin-testinal	4678	364	130	61.0 (17.25)	119 female, 245 male
**TCGA-LUAD**	Lung adenocar-cinoma	Thoracic	4984	490	179	66.0 (13.00)	266 female, 224 male
**TCGA-LUSC**	Lung squamous cell carcinoma	Thoracic	5391	488	211	68.0 (11.00)	127 female, 361 male
**TCGA-MESO**	Mesotheli-oma	Thoracic	3731	84	72	64.0 (12.00)	15 female, 69 male
**TCGA-OV**	Ovarian serous cystadeno-carcinoma	Gyneco-logic	4696	372	229	59.0 (17.00)	372 female
**TCGA-PAAD**	Pancreatic adenocar-cinoma	Gastroin-testinal	4489	176	92	65.0 (16.00)	80 female, 96 male
**TCGA-READ**	Rectum adenocar-cinoma	Gastroin-testinal	3137	156	26	65.0 (15.00)	68 female, 88 male
**TCGA-SARC**	Sarcoma	Soft tissue	4503	256	98	60.5 (17.25)	139 female, 117 male

**TCGA-SKCM**	Skin cutaneous melanoma	Melano-cytic	3116	98	28	63.5 (18.75)	40 female, 58 male
**TCGA-STAD**	Stomach adenocar-cinoma	Gastroin-testinal	4864	344	143	67.0 (14.00)	123 female, 221 male
**TCGA-UCEC**	Uterine corpus endometrial carcinoma	Gyneco-logic	4723	537	90	64.0 (14.00)	537 female
**TCGA-UCS**	Uterine carcinosar-coma	Soft tissue	3436	54	33	68.5 (13.50)	54 female
**TCGA-UVM**	Uveal melanoma	Melano-cytic	2062	80	23	61.5 (23.25)	35 female, 45 male
**TCGA-CHOL**	Cholangio-carcinoma	Gastroin-testinal	N/A	36	18	66.5 (15.50)	20 female, 16 male
**TCGA-DLBC**	Lymphoid neoplasm diffuse large B-cell lymphoma	Hemato-logic and lymphatic malignan-cies	N/A	47	9	58.0 (21.00)	26 female, 21 male
**TCGA-KICH**	Kidney chromo-phobe	Urologic	N/A	64	9	50.0 (19.25)	26 female, 38 male
**TCGA-PCPG**	Pheochro-mocytoma and paragangli-oma	Neural-crest derived	N/A	178	6	46.0 (23.50)	101 female, 77 male
**TCGA-PRAD**	Prostate adenocar-cinoma	Urologic	N/A	493	10	61.0 (10.00)	493 male
**TCGA-TGCT**	Testicular germ cell tumors	Urologic	N/A	134	4	31.0 (11.00)	134 male
**TCGA-THCA**	Thyroid carcinoma	Endocrine	N/A	501	16	46.0 (23.00)	366 female, 135 male
**TCGA-THYM**	Thymoma	Hemato-logic and lymphatic malignan-cies	N/A	118	9	59.5 (20.50)	56 female, 62 male

Table S2. The 103 survival module genes, Related to Figure 4. Each of the 103 module genes identified in the network propagation and module identification steps is annotated with its original feature importance weight derived from pan-cancer XGBoost training, the weight and corresponding p-value after network propagation and the type of the gene (seed gene, other pan-cancer feature, or inferred during network propagation). * after a gene name indicates known cancer genes and ** indicates candidate cancer genes according to NCG 6.0 (Repana et al., 2019).

Gene	Original Feature Importance	Network Propagation Weight	Network Propagation P-value	Type of Gene
BCHE	282.92	227.86	0.01	Seed gene
TMEM30B	272.23	221.27	0.01	Seed gene
INS	235.63	206.38	0.01	Seed gene
TREM1	231.79	185.75	0.01	Seed gene
ADRA1D	210.45	168.53	0.01	Seed gene
SEMA7A	190.95	154.80	0.01	Seed gene
CDH10*	177.00	143.35	0.01	Seed gene
SPP1	164.45	136.74	0.01	Seed gene
APP	50.89	135.16	0.01	Pan-cancer feature
BTLA**	158.03	127.95	0.01	Seed gene
SCG5	153.32	123.76	0.01	Seed gene
PLAU	112.53	95.14	0.01	Pan-cancer feature
NCAM1	0.00	77.62	0.01	Inferred gene
RBL2	0.00	72.48	0.01	Inferred gene
TEAD1	10.50	71.72	0.01	Pan-cancer feature
PLAUR	2.27	65.34	0.01	Pan-cancer feature
FYN**	1.53	56.14	0.01	Pan-cancer feature
FBLN1	0.00	54.58	0.01	Inferred gene
A2M	0.00	52.55	0.01	Inferred gene
COLQ	0.00	45.69	0.01	Inferred gene
CDK5	4.38	43.72	0.01	Pan-cancer feature
SGCD	53.26	43.37	0.01	Pan-cancer feature
PLA2G4A	5.90	42.16	0.01	Pan-cancer feature
CAV1	0.00	40.55	0.01	Inferred gene
TP63*	0.00	40.54	0.01	Inferred gene
TMEM25	42.74	38.56	0.01	Pan-cancer feature
ITGA3	39.06	33.29	0.01	Pan-cancer feature

PLG	0.00	32.80	0.01	Inferred gene
SFN	0.00	32.00	0.01	Inferred gene
TLR4**	0.00	31.18	0.01	Inferred gene
ATP8B2**	0.00	29.59	0.01	Inferred gene
FKBP1A	15.99	29.09	0.01	Pan-cancer feature
EPHA2**	7.48	28.90	0.01	Pan-cancer feature
DCTN1*	0.00	28.64	0.01	Inferred gene
MMP14	7.40	28.13	0.01	Pan-cancer feature
PCSK2	1.78	27.47	0.01	Pan-cancer feature
ERBB4*	0.00	26.77	0.01	Inferred gene
MMP3	4.19	26.65	0.01	Pan-cancer feature
TNFRSF14*	0.00	23.19	0.01	Inferred gene
FGF2**	11.51	23.13	0.01	Pan-cancer feature
LUZP1	0.00	22.71	0.01	Inferred gene
CDH6	8.46	22.23	0.01	Pan-cancer feature
AGL	0.00	22.13	0.01	Inferred gene
PRMT6	0.00	21.52	0.01	Inferred gene
IGSF21	18.58	21.05	0.01	Pan-cancer feature
GLYR1**	0.00	20.84	0.01	Inferred gene
MMP1	13.14	20.74	0.01	Pan-cancer feature
TGFBR2*	0.00	20.68	0.01	Inferred gene
JAK2*	0.00	20.39	0.01	Inferred gene
LRP2**	8.62	19.98	0.01	Pan-cancer feature
PICALM*	0.00	19.34	0.01	Inferred gene
RAB27B	19.94	19.27	0.01	Pan-cancer feature
ADRA1A**	0.00	18.64	0.01	Inferred gene
RPS6KA3**	0.00	18.51	0.01	Inferred gene
EIF4G3	0.00	18.05	0.01	Inferred gene
DPYSL3	10.86	18.05	0.01	Pan-cancer feature
HSF2BP	0.00	17.93	0.01	Inferred gene
IGF2**	0.00	17.24	0.01	Inferred gene
GNAI3	0.00	17.14	0.01	Inferred gene
COL17A1	2.45	16.91	0.01	Pan-cancer feature
SERPINE1	558.51	451.64	0.02	Seed gene

VTN	397.78	327.91	0.02	Seed gene
LARGE2	365.16	292.92	0.02	Seed gene
TGFB1	339.00	282.17	0.02	Seed gene
PAEP	256.85	205.82	0.02	Seed gene
CLDN4	240.49	192.73	0.02	Seed gene
IGFBP1	221.18	177.40	0.02	Seed gene
ADAM9	170.62	138.16	0.02	Seed gene
DPYSL5	161.96	129.96	0.02	Seed gene
FLNC	488.05	399.73	0.03	Seed gene
UNC13D	207.33	166.16	0.04	Seed gene
PTX3	405.79	326.90	0.05	Seed gene
PIK3R3	870.79	701.96	0.06	Seed gene
DKK1	328.54	262.89	0.06	Seed gene
MLC1	243.16	194.63	0.06	Seed gene
TIMP4	218.82	175.46	0.07	Seed gene
EYA4**	203.69	162.99	0.10	Seed gene
S100A10	164.35	134.44	0.12	Seed gene
ST8SIA3	476.24	381.05	0.13	Seed gene
ARHGEF3	157.88	126.33	0.14	Seed gene
LAD1	278.45	222.81	0.15	Seed gene
PLEC**	281.59	229.50	0.16	Seed gene
DLG3**	376.41	303.90	0.17	Seed gene
HJURP	348.03	278.72	0.18	Seed gene
BARX1	454.28	363.58	0.19	Seed gene
CENPA	362.38	298.74	0.19	Seed gene
BCAT1	244.63	195.73	0.21	Seed gene
VGLL2	383.43	306.77	0.23	Seed gene
CCDC88C	167.97	134.40	0.29	Seed gene
IRF6**	432.21	345.81	0.32	Seed gene
PLA2G5	209.22	167.38	0.35	Seed gene
FRK	155.23	124.26	0.40	Seed gene
KIF2C	158.16	128.35	0.41	Seed gene
CDK6*	173.38	142.07	0.46	Seed gene
LBX1	438.19	350.56	0.51	Seed gene

CDK5R2	238.29	190.64	0.52	Seed gene
ZNF557	193.75	155.01	0.55	Seed gene
CDC20	441.45	358.13	0.83	Seed gene
IDO1	377.05	301.65	0.86	Seed gene
RPS6KA5	152.61	123.09	0.87	Seed gene
PGR*	246.63	198.55	0.99	Seed gene
IGF2BP2*	241.03	193.01	0.99	Seed gene
ESR1*	240.89	215.59	1.00	Seed gene

Table S3. Top 15 cancer-relevant upstream regulators computed with QIAGEN Ingenuity Pathway Analysis (IPA) (Krämer et al., 2014), Related to Figure 4. Upstream regulator: gene name of annotated cancer driver gene or potential cancer driver gene (Repana et al., 2019) as upstream regulator of the pan-cancer survival network; molecule type: molecule type of the upstream regulator; p-value of overlap: Fisher test p-value for over-representation of network genes in target set of upstream regulator; target molecules in dataset: network module genes downstream of the upstream regulator

Upstream Regulator	Molecule Type	P-value of Overlap	Target Molecules in Dataset
JUN	Transcription regulator	1.20E-13	*A2M, APP, CAV1, CDC20, DKK1, FGF2, FLNC, IGFBP1, MMP1, MMP3, NCAM1, PGR, PLA2G4A, PLAU, PLAUR, PTX3, S100A10, SERPINE1, SPP1, TGFB1*
TNF	Cytokine	1.88E-12	*A2M, APP, CAV1, CLDN4, COLQ, DKK1, EPHA2, ESR1, FGF2, FYN, GNAI3, IDO1, IGF2, IGFBP1, INS, LAD1, MMP1, MMP14, MMP3, NCAM1, PLA2G4A, PLA2G5, PLAU, PLAUR, PTX3, S100A10, SERPINE1, SPP1, TGFB1, TGFBR2, TIMP4, TLR4, TP63, TREM1*
IL1B	Cytokine	7.12E-12	*A2M, APP, ESR1, FGF2, IDO1, IGFBP1, INS, ITGA3, MMP1, MMP14, MMP3, PCSK2, PGR, PLA2G4A, PLA2G5, PLAU, PTX3, S100A10, SERPINE1, SPP1, TGFB1, TGFBR2, TIMP4, TLR4, TREM1*
TP53	Transcription regulator	1.63E-11	*A2M, ADRA1A, APP, BCAT1, CAV1, CDC20, CDH10, CENPA, DKK1, EIF4G3, EPHA2, ESR1, EYA4, FGF2, FKBP1A, FYN, HJURP, IGF2, IGF2BP2, INS, MMP1, MMP3, PGR, PIK3R3, PLAU, PLAUR, RBL2, SERPINE1, SFN, SPP1, TGFB1, TGFBR2, TP63*

IL1A	Cytokine	1.37E-10	*APP, FGF2, MMP1, MMP14, MMP3, PLA2G4A, PLAU, PTX3, RBL2, S100A10, SERPINE1, SPP1, TGFB1*
FGF2	Growth factor	1.28E-09	*AGL, CAV1, DKK1, FGF2, IGF2, ITGA3, MMP1, MMP3, PCSK2, PLAU, PLAUR, S100A10, SERPINE1, SPP1, TGFB1*
MAP3K1	Kinase	5.30E-09	*MMP3, PGR, PLA2G4A, PLAU, PLAUR, SERPINE1, TGFB1*
EGFR	Kinase	1.03E-08	*APP, CAV1, CDK6, EPHA2, ERBB4, ESR1, IGF2, MMP1, MMP14, MMP3, PLA2G4A, PLAU, PLAUR, SEMA7A, SERPINE1*
STAT3	Transcription regulator	3.37E-08	*A2M, DKK1, ESR1, FGF2, IGFBP1, JAK2, LRP2, MMP1, MMP3, PGR, PLA2G4A, PLAU, PLAUR, SERPINE1, SPP1, TGFB1*
HRAS	Enzyme	8.45E-08	*A2M, APP, CAV1, EIF4G3, FGF2, IGF2, MMP1, MMP14, MMP3, PLA2G4A, PLAU, PLAUR, SERPINE1, SPP1, TGFB1, TP63*
CDH1	Other	1.32E-07	*ERBB4, MMP1, MMP14, MMP3, NCAM1, PLAUR, TGFB1*
AKT1	Kinase	1.36E-07	*ESR1, FGF2, IGF2, IGFBP1, MMP14, PGR, PLA2G4A, PLG, SERPINE1, SPP1, TP63*
PTEN	Phosphatase	1.38E-07	*CDC20, CDK6, ESR1, IGF2, LAD1, MMP14, MMP3, NCAM1, PLAU, PLEC, RBL2, SERPINE1, SPP1, TGFB1, TGFBR2, TNFRSF14*
FOXO1	Transcription regulator	2.36E-07	*A2M, CAV1, FYN, IGFBP1, INS, ITGA3, MMP1, MMP3, RBL2, RPS6KA3, SERPINE1, SFN, TGFB1*
SMARCA4	Transcription regulator	2.49E-07	*A2M, GNAI3, IRF6, ITGA3, MMP1, PAEP, PLAUR, PTX3, SCG5, SEMA7A, SERPINE1, SPP1, TNFRSF14, TREM1, UNC13D*
ERBB2	Kinase	2.91E-07	*CDC20, CDK6, CENPA, CLDN4, ERBB4, ESR1, IGF2, IRF6, MMP1, MMP14, MMP3, PLAU, PLAUR, RBL2, SCG5, SERPINE1, TP63, TREM1*
PRKCB	Kinase	3.05E-07	*APP, FGF2, INS, SERPINE1, TGFB1, TGFBR2*
ITGAV	Transmembrane receptor	3.43E-07	*MMP1, PLAU, SERPINE1, TGFB1, VTN*
CD36	Transmembrane receptor	3.77E-07	*FGF2, MMP1, MMP14, MMP3, PLAU, PLAUR, SERPINE1*
TP73	Transcription regulator	4.29E-07	*CDC20, EPHA2, FGF2, MMP14, NCAM1, PIK3R3, PLAU, SERPINE1, SFN, SPP1, TGFB1, TIMP4*

CDKN1A	Kinase	4.90E-07	*APP, CDC20, HJURP, KIF2C, MMP1, MMP3, RBL2, SERPINE1, TP63, TREM1*
FGFR1	Kinase	4.94E-07	*FGF2, MMP1, MMP14, MMP3, PLAU, PLAUR, SFN*
FOXO3	Transcription regulator	5.05E-07	*CAV1, CDC20, ESR1, FYN, IGFBP1, KIF2C, PLAU, RBL2, SERPINE1, TGFB1, TP63*
NRG1	Growth factor	5.07E-07	*CAV1, CDC20, EPHA2, FGF2, IGF2, PGR, PLAU, PLAUR, PLG, SERPINE1*
HGF	Growth factor	5.24E-07	*A2M, CAV1, CDC20, KIF2C, MMP1, MMP14, PLAU, PLAUR, SERPINE1, SPP1, TGFB1, TGFBR2, TP63*
ETV4	Transcription regulator	6.33E-07	*CAV1, MMP14, PLEC, SPP1, TGFBR2*
MYC	Transcription regulator	7.56E-07	*APP, BCAT1, CAV1, CDC20, CDK6, DKK1, EPHA2, GLYR1, INS, IRF6, ITGA3, NCAM1, PLAU, PLAUR, S100A10, SERPINE1, SPP1, TEAD1, TGFB1, TGFBR2*
RAF1	Kinase	7.95E-07	*ESR1, INS, LAD1, MMP1, MMP3, PLAU, PLAUR, PLEC, RPS6KA5*
ETS1	Transcription regulator	9.96E-07	*CAV1, CDK6, MMP1, MMP3, PGR, PLAU, SERPINE1, SPP1, TGFBR2*
NFKBIA	Transcription regulator	1.01E-06	*A2M, FGF2, IGF2, ITGA3, MMP1, MMP14, MMP3, PICALM, PLAU, PTX3, TGFB1, TLR4*
NR3C1	Ligand-dependent nuclear receptor	1.07E-06	*A2M, ADRA1D, APP, ARHGEF3, CAV1, IGFBP1, LAD1, MMP1, PIK3R3, PLA2G4A, SERPINE1, SPP1, TGFB1, TIMP4, VGLL2*
GLIS2	Transcription regulator	1.24E-06	*INS, MMP14, SERPINE1, TGFB1*
ESR1	Ligand-dependent nuclear receptor	1.65E-06	*BCAT1, CAV1, CDK5, CDK6, CENPA, CLDN4, ERBB4, ESR1, FBLN1, HSF2BP, IGF2, JAK2, MMP1, PGR, PLAU, PLAUR, PTX3, RBL2, SERPINE1, SPP1, TGFB1*
NAB2	Transcription regulator	1.94E-06	*FGF2, MMP3, PLAU, TGFB1*
TGFA	Growth factor	2.17E-06	*ESR1, PLA2G4A, PLA2G5, S100A10, SERPINE1, TGFB1*
TP63	Transcription regulator	2.37E-06	*CDK6, DKK1, EPHA2, ITGA3, MMP14, PIK3R3, PLAU, SERPINE1, SFN, TGFB1, TGFBR2, TP63*
DLC1	Other	3.28E-06	*CDK6, S100A10, SERPINE1*
AREG	Growth factor	3.29E-06	*CDC20, CENPA, HJURP, MMP1, PLAU, PTX3*
MAP2K1	Kinase	3.39E-06	*DKK1, FGF2, INS, MMP1, MMP14, MMP3, PLA2G4A, PLAUR*

PPARG	Ligand-dependent nuclear receptor	3.82E-06	*APP, CAV1, CDK6, IGFBP1, INS, MMP14, MMP3, SERPINE1, SPP1, TGFBR2, TIMP4, TLR4*
NOTCH1	Transcription regulator	4.45E-06	*DKK1, FGF2, MMP1, MMP3, SERPINE1, SPP1, TGFB1, TGFBR2, TP63*
ZEB1	Transcription regulator	4.54E-06	*MMP1, PLAU, RBL2, S100A10, SERPINE1, TP63*
TGFBR2	Kinase	4.57E-06	*FGF2, MMP14, MMP3, RBL2, SERPINE1, SPP1, TGFB1, TGFBR2*
TERT	Enzyme	4.91E-06	*CAV1, COLQ, FGF2, MMP1, MMP14, MMP3, SPP1*
ABL2	Kinase	5.23E-06	*MMP1, MMP14, MMP3*
ERG	Transcription regulator	5.66E-06	*FLNC, FYN, MMP1, MMP3, PLAU, PLAUR, SPP1, TGFBR2*
IKBKB	Kinase	7.93E-06	*FYN, MMP1, MMP3, PLA2G4A, PLAU, PTX3, TGFB1, TGFBR2, TP63*
